# Rosuvastatin revert memory impairment and anxiogenic-like effect in mice infected with the chronic ME-49 strain of *Toxoplasma gondii*

**DOI:** 10.1371/journal.pone.0250079

**Published:** 2021-04-15

**Authors:** Fernanda Ferreira Evangelista, Willian Costa-Ferreira, Francini Martini Mantelo, Lucimara Fátima Beletini, Amanda Hinobu de Souza, Priscilla de Laet Sant’Ana, Keller Karla de Lima, Carlos Cesar Crestani, Ana Lúcia Falavigna-Guilherme

**Affiliations:** 1 Health Sciences Department, State University of Maringá (UEM), Maringá, PR, Brazil; 2 School of Pharmaceutical Sciences, São Paulo State University (UNESP), Araraquara, SP, Brazil; 3 Pharmaceutical Sciences Program, State University of Maringá (UEM), Maringá, PR, Brazil; 4 Resident of Obstetric Nursing of the Municipal Health Authority of Apucarana, Apucarana, PR, Brazil; Golestan University of Medical Sciences and Health Services, ISLAMIC REPUBLIC OF IRAN

## Abstract

The aim of this study was to investigate the effect of rosuvastatin treatment on memory impairment, and anxiogenic-like effects in mice chronically infected with *Toxoplasma gondii*. For this, Balb/c mice were infected orally with chronic ME-49 strain of *Toxoplasma gondii*. Oral treatment with rosuvastatin (40mg/kg/day) started on the 51^st^ day post-infection and was performed daily for 21 days. After completion of treatment, anxiety-like effects and locomotion were investigated in the open field (OF) test, whereas novel object recognition (NOR) test was used for evaluation of short- and long-term memory. At the end of the experiments, the brain was collected for *Toxoplasma gondii* DNA quantification and histopathological analysis. Infection with ME-49 strain decreased the time spent in the center of OF, indicating an anxiogenic effect, without affecting total and peripheral locomotion. Rosuvastatin treatment inhibited the change in the center time. Besides, pharmacological treatment increased total and central locomotion in both non-infected and infected animals. Infection also impaired both short- and long-term memory in the NOR test, and these effects were reverted by rosuvastatin treatment. In addition to effects in behavioral changes, rosuvastatin also reduced parasite load in the brain and attenuated signs of brain inflammation such as perivascular cuffs, inflammatory cell infiltration and tissue damage. These findings indicate for the first time the efficacy of rosuvastatin in treatment of memory impairment and anxiogenic effect evoked by infection with *Toxoplasma gondii*. These effects might be mediated by reduced cyst load, which in turn decrease inflammation and damage in the brain.

## Introduction

Toxoplasmosis is a zoonotic infection caused by intracellular protozoan *Toxoplasma gondii (T*. *gondii)* [1]. It is estimated that one-third of the world’s population is infected by this parasite [2–4]. Felids are the definitive hosts that eliminate millions of oocysts (sporozoites) in their feces. These forms spread in the environment contaminating water, soil, fruits and vegetables; which in turn can infect animals and humans [1]. Besides, the infection to humans can happen through the ingestion of undercooked meat containing viable tissue cysts with bradyzoites [1]. Congenital transmission is another important pathway of infection when the *T*. *gondii* tachyzoites (acute phase) of the maternal host reach the placenta and the fetus [5]. Toxoplasmosis is asymptomatic in the majority of the immunocompetent patients [3], but immunocompromised patients can experience severe neurologic, ocular, pulmonary and disseminated diseases [6].

The immune responses to control parasite multiplication cannot eradicate the infection. Thereby, the chronic phase is marked by the presence of cysts with bradyzoites that remain in tissues, mainly in the central nervous system (CNS), where there is intense parasite tropism in human and rodent hosts [1]. While most CNS pathogens are strongly harmful, causing serious damage and symptoms, including host death, *T*. *gondii* seem to remain latent in a variety of tissues of immunocompetent individuals, especially CNS, skeletal and cardiac muscle [7]. Despite the apparent absence of manifestations in chronically infected immunocompetent individuals, some studies have demonstrated an association between chronic toxoplasmosis and psychiatric disorders in humans and behavioral changes in animal hosts. Some studies have suggested that chronic infection with *T*. *gondii* in the CNS may be associated with neurological changes and psychiatric disorders in humans such as schizophrenia, personality changes, dementia and suicidal tendencies (depression) [8–13]. In rodents, the chronic infection promotes memory impairment and behavioral changes including increase in anxiety, schizophrenia and depressive-like behaviors; and greater exposure and attraction to predators [12, 14].

Therapeutic options for this zoonosis are still scarce and do not act in the chronic phase of infection [15]. The most efficient therapeutic scheme is the combination of pyrimethamine, sulfadiazine and folinic acid; which block *T*. *gondii* tachyzoites proliferation by inhibiting folate synthesis [16, 17]. However, this treatment is not effective against bradyzoites inside of tissue cysts and has important side effects. Therefore, the treatment in the chronic phase is difficult, even to prevent the recurrence of retinochoroiditis [15, 18–20]. In view of the high prevalence of toxoplasmosis and the difficulty to treat the infection during the chronic phase, other compounds have been tested as therapeutic options against *T*. *gondii*. Statins, drugs often used to lower blood cholesterol levels in humans, have been demonstrated to reduce tachyzoites replication *in vitro* and *in vivo* [21–26], and rosuvastatin reduced parasite load in brain of mice chronically infected [26]. Steroids and isoprenoids are essential lipids for *T*. *gondii* metabolism [27] and interference in the initial protozoan isoprenoid biosynthesis processes has been proposed as a mechanism involved in antiparasitic effects of statins [22, 23]. However, despite these pieces of evidence, it remains to be determined the effect of rosuvastatin treatment on memory impairment and behavioral changes related to anxiety during *T*. *gondii* infection. Therefore, the aim of this work was to investigate the effect of rosuvastatin treatment on impairment of short- and long-term memory and increased anxiety-like behaviors in mice chronically infected with chronic ME-49 strain of *T*. *gondii*.

## Material and methods

### Animals

Forty female Balb/c mice at three weeks of age were used in this study. The animals were obtained from the animal breeding facility of the Maringá State University-UEM (Maringá, PR, Brazil). All animals were grouped in standard laboratory cages with sawdust and free access to food and water. The holding room had a 12:12 h light-dark cycle (lights on at 07:00 a.m.), with an average temperature of 20–25°C and humidity (60 ± 10%). All procedures in this study were conducted in accordance with the recommendations of the institutional guidelines for animal ethics and were approved by the *Comitê de Ética na Utilização de Animais* (CEUA-UEM) (approval # 5654290317).

### *Toxoplasma gondii* strain

*T*. *gondii* ME-49 strain (genotype II) was used to induce experimental chronic toxoplasmosis. The parasite cysts were obtained from brains of chronically infected mice. For this, the mice brain was mechanically homogenized in saline after animal sacrifice. Number of cysts in the homogenate was counted using light microscopy [26]. Mice were infected orally with approximately 25 cysts from brain homogenate diluted in 0.5 mL saline by oral gavage. Control mice received 0.5 mL of saline orally.

### Experimental design

Experimental tests included groups orally infected (p.o.) with *T*. *gondii* ME-49 strain (infected groups) and non-infected groups (control groups). After 50 days of infection, on the 51^st^ day, infected and control groups were subjected to daily administration of either rosuvastatin or saline solution for 21 days. The animals were randomly assigned to experimental groups. Rosuvastatin was given daily (at 8:00 a.m.) by gavage at the dose of 40mg/kg [26, 28].

The locomotor activity and anxiety-like behavior were evaluated using the open field (OF) test on the 72^nd^ day, 24 h after the last treatment. Twenty-four hours later (i.e.,73^rd^ day), animals of all experimental groups were allowed to habituate for five min to the apparatus wherein the novel object recognition (NOR) test was performed (the same apparatus used in the OF test). Twenty-four hours later (74^th^ day), the animals were subjected to procedures for evaluation of short-term memory. The long-term memory was assessed 24 h later (i.e., 75^th^ day) in the same apparatus. After the behavioral tests, the animals were euthanized, and the brain was collected for histopathological analysis.

### Open field test

The OF test was used for evaluation of anxiety-like behaviors and locomotion [29–31]. It consisted of enclosure area with 30 cm diameter surrounded by opaque walls (30 cm height), with its circular floor divided by lines into four central and eight peripheral quadrants of equivalent area. The central area was defined as an exposed field and is referred to as “center”. Mice were individually placed in the middle of the arena and were allowed to explore freely the apparatus for 5 min. Analysis included measures of the distance travelled in central (central locomotion) and peripheral area (peripheral locomotion), as well as the total distance travelled (i.e., center + periphery) (total locomotion) [32, 33].

All sessions were videotaped (Webcam LifeCam Cinema HD 720p Microsoft® using software Microsoft LifeCam version 3.22) and analysis was performed in a blinded manner using the behavioral tracking software ANY-maze (Stoelting, Wood Dale, IL, USA).

### Novel object recognition test

The NOR test was used for evaluation of the learning performance and non-emotional memory. Initially, animals were allowed to habituate for five min to the apparatus wherein the NOR test was performed (the same apparatus used in the OF test). Twenty-four hours later, the animals were subjected to procedures for evaluation of short-term memory. At this stage, objects A and A’ (a pair of transparent square plastic bottles) were centered at the ends of the apparatus, 10 cm from the walls. Ten min later, the object A’ was replaced by a rectangular object B, and the short-term memory was assessed. The long-term memory was evaluated 24 h later in the same apparatus by changing the object B by a new object, an orange sphere called object C. The same animals were evaluated for short- and long-term memory [32, 33].

For evaluation of short- and long-term memory, the time the animals explored each object was recorded (Webcam LifeCam Cinema HD 720p Microsoft® using software Microsoft LifeCam version 3.22), and the exploration was blindly analyzed using the software X-PloRat (version 2005, 1.1.0). For each object, the interaction period was defined as the time while the animal remained in physical contact with the object. Data were presented as the discrimination index, which was determined by time spent on the new object divided by the time spent on both objects [32, 33].

### Histopathological analysis

The animals were euthanized with inhaled isoflurane and cervical dislocation, and the brain was collected and divided in two parts. One part was used for analysis of parasite load and the other one for histopathological analysis. The latter procedure was performed by fixing the brain tissue in 10% formalin in PBS at pH 7.2 for 18–24 h, and then transferred and maintained in 70% ethanol until tissue processing. The tissues were processed by dehydration and paraffin embedding. Three 5μm histological sections (non-continuous sequence) from each sample were prepared and stained with Harris haematoxylin and eosin (HE) techniques. The sections were examined with a light microscope (Optcam O500R) (with 10, 20, 40 and 100x objectives). The pathological lesions in brain tissue were qualitatively classified for the presence of the following parameters: meningitis, perivascular cuffs, inflammatory cell infiltration, necrosis, haemorrhage and gliosis [26, 34].

### Molecular diagnosis of *T*. *gondii*

The frozen brain part of each mouse was macerated with saline solution and the total genomic DNA was extracted from 100uL using a commercial kit ReliaPrep^TM^ gDNA Tissue Miniprep System (Promega, USA), following the manufacturer’s recommendations. Total DNA concentration was determined with a NanoDrop spectrophotometer (Thermo Fisher Scientific, US). Quantitative polymerase chain reaction (qPCR) was performed in duplicate with 50 ng of the total extracted DNA using the QuantiNova SYBR™ Green PCR Kit (Qiagen, Hilden, Germany), according to the manufacturer’s instructions. Primers B1 (B22–B23; forward: 5′-AACGGGCGAGTAGCACC TGAGGAGA-3′ and reverse: 5′-TGGGTCTACGTCGATGGC ATGACAAC-3′) were used in order to amplify a 115 base pairs (bp) sequence in *T*. *gondii* (Burg et al., 1989). In each reaction, a negative control (mixture without DNA) and a positive control (DNA extracted from ME-49 strain) were processed. The reaction was performed with a LightCycler 96 (Roche Diagnostics, Mannheim, Germany) thermal cycler using reaction conditions recommended by the manufacturer: 2 min at 95°C, followed by 10 min at 95°C and 50 cycles at 60°C for 30 s. For the final analysis, a denaturation curve was performed from 60 to 97°C, followed by electrophoresis of the products in order to ensure there were no nonspecific amplifications or dimers. The standard curve was processed using serially diluted DNA in Milli-Q water [35]. The DNA quantity (ng) for each sample was deduced from standard curves and then converted in equivalent parasites (Eq. parasite) [36].

### Data analysis

All data were analyzed using the software GraphPad Prism version 7.0 (GraphPad Software Inc., La Jolla, CA, USA). The data of parasite load in the brain was analyzed using one-way ANOVA. The results of the behavioral analysis were analyzed using two-way ANOVA, with infection (infected x non-infected) and treatment (vehicle x rosuvastatin) as independent factors. When differences were identified by one and two-way ANOVA, Bonferroni *post-hoc* test was performed to assess specific differences between the experimental groups. P<0.05 was assumed as significant.

## Results

### Effect of infection with *T*. *gondii* ME-49 strain and treatment with rosuvastatin in locomotion and anxiety-like behaviors

**Fig 1:**
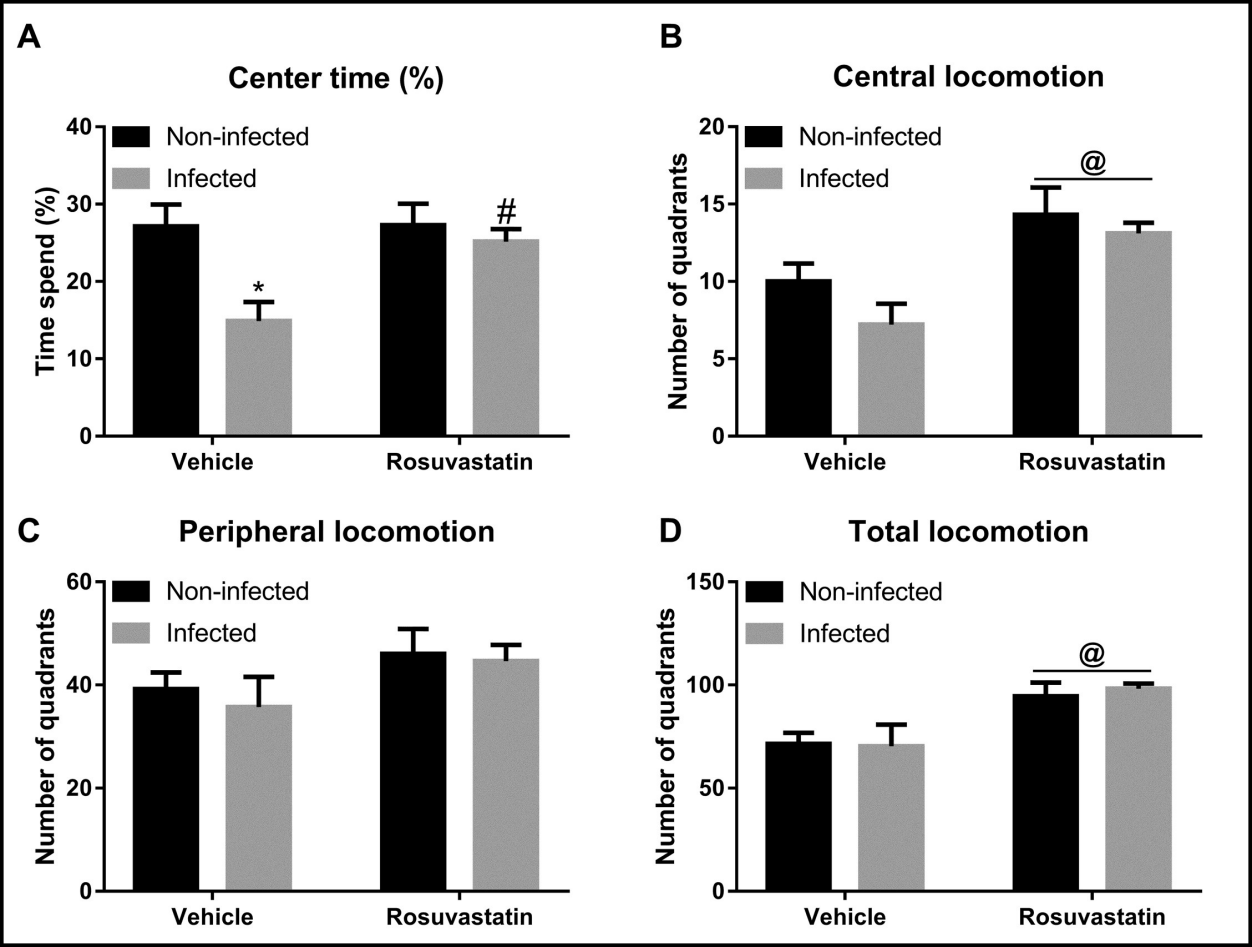
Behavioral analysis in the Open Field (OF) test in animals non-infected and infected with *Toxoplasma gondii* ME-49 strain and treated with rosuvastatin or vehicle. The bars represent the mean ± SEM. *P<0.05 vs respective non-infected group, # P<0.05 vs respective vehicle group, @ P<0.05 indicating main effect of treatment. Two-way ANOVA followed by Bonferroni *post-hoc* test (n = 10/group).

Analysis of time spent in center of the OF (Fig 1) indicated main effect of infection (F_(1,36)_ = 8.37, P < 0.01) and treatment (F_(1,36)_ = 4.41, P < 0.05) as well as an infection x treatment interaction (F _(1,36)_ = 4.15, P < 0.05) (Fig 1A). *Post-hoc* analysis revealed that infected mice treated with vehicle had significantly reduced time spent in the center of the apparatus in relation to non-infected animals treated with vehicle (P < 0.01). Besides, rosuvastatin treatment completely inhibited this change (P < 0.05) (Fig 1A).

Analysis of peripheral locomotion did not indicate effect of either infection (F _(1,36)_ = 0.31, P > 0.05) and treatment (F _(1,36)_ = 3.14, P > 0.05) as well as an infection x treatment interaction (F _(1,36)_ = 0.06, P > 0.05) (Fig 1C). Nevertheless, evaluation of central and total locomotion indicated effect of treatment (central: F _(1,36)_ = 15.24, P < 0.001; total: F _(1,36)_ = 13.86, P < 0.001), but without main influence of infection (central: F _(1,36)_ = 2.34, P > 0.5; total: F _(1,36)_ = 0.03, P > 0.5) and infection x treatment interaction (central: F _(1,36)_ = 0.37, P > 0.5; total: F _(1,36)_ = 0.12, P > 0.5) (Fig 1B and 1D).

### Effect of infection with *T*. *gondii* ME-49 strain and treatment with rosuvastatin in short- and long-term memory

**Fig 2:**
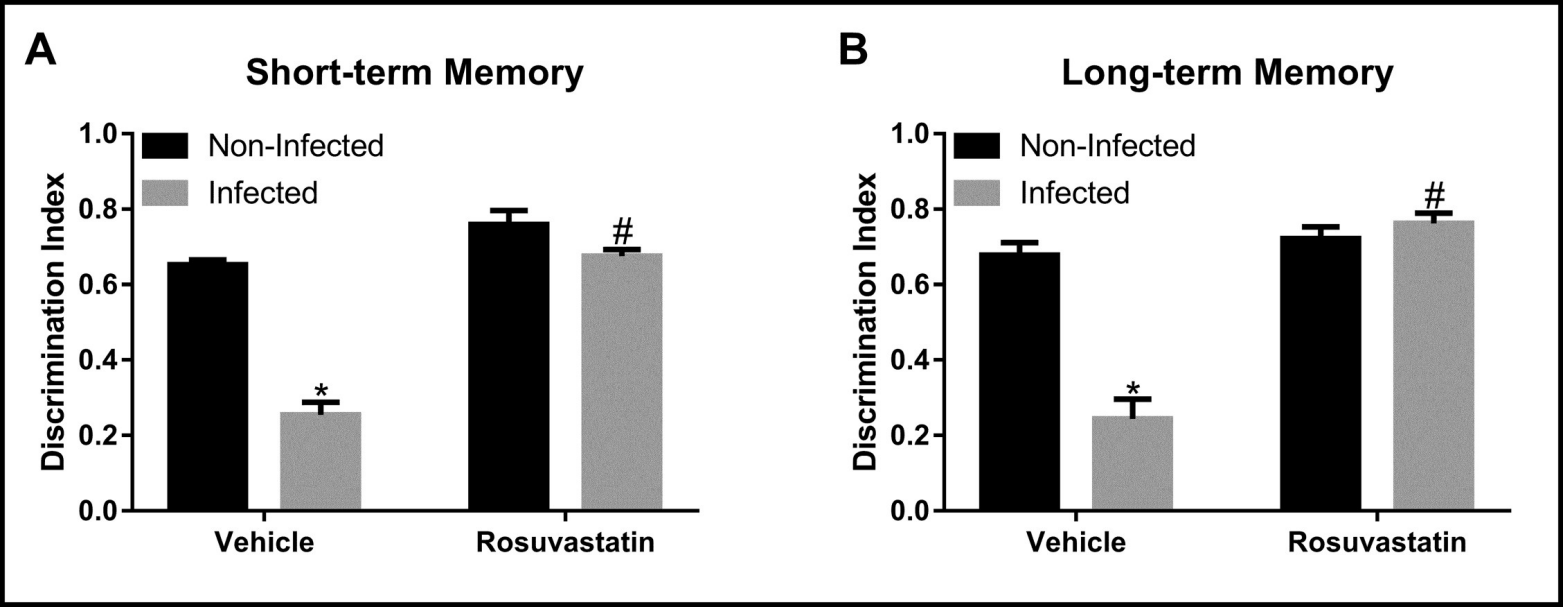
Cognitive non-emotional performance in the Novel Object Recognition (NOR) test in animals non-infected and infected with *Toxoplasma gondii* ME-49 strain and treated with rosuvastatin or vehicle. The bars represent the mean ± SEM. *P < 0.05 vs respective non-infected group, #P < 0.05 vs respective vehicle group. Two-way ANOVA followed by Bonferroni *post-hoc* test (n = 10/group).

Analysis of short- and long-term memory indicated main effects of infection (short-term: F_(1,36)_ = 80.27, P < 0.0001; long-term: F_(1,36)_ = 27.69, P < 0.0001) and treatment (short-term: F_(1,36)_ = 95.75, P<0.001; long-term: F_(1,36)_ = 56.49, P < 0.0001) as well as an infection x treatment interaction (short-term: F_(1,36)_ = 3.87, P<0.001; long-term: F_(1,36)_ = 40.15, P < 0.0001) (Fig 2). *Post hoc* analysis revealed that infection with *T*. *gondii* ME-49 strain reduced exploration of new object in relation to non-infected mice in both short- and long-term memory (P < 0.0001) (Fig 2), which indicates a memory and learning impairment. Rosuvastatin treatment inhibited the short- and long-term memory impairment evoked by infection (P < 0.0001) (Fig 2).

### Effect of treatment with rosuvastatin in parasite load and histopathological changes in the brain evoked by infection with *T*. *gondii* ME-49 strain

**Fig 3:**
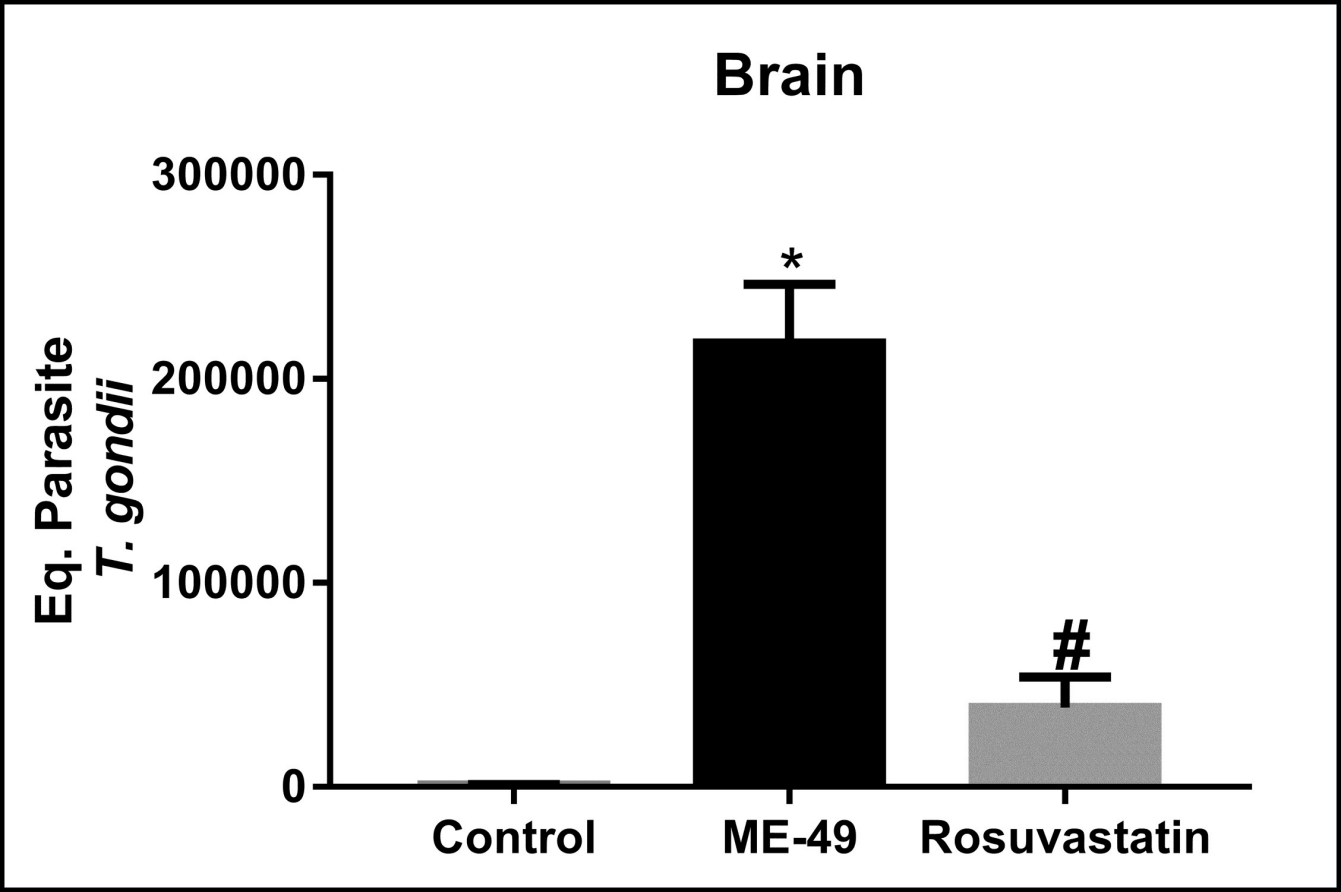
Parasite load in the brain of mice infected with the *Toxoplasma gondii* ME49 strain (in equivalent parasites). Control: non-infected animals; ME-49: infected animals treated with vehicle; The bars represent the mean ± SEM. *P < 0.05 vs control group, #P < 0.05 vs ME-49 group. One-way ANOVA followed by Bonferroni *post-hoc* test (n = 10/group).

*T*. *gondii* DNA was detected in the brains of all mice infected with the ME49 strain of *T*. *gondii*. Quantitative PCR (qPCR) analysis demonstrated that treatment with rosuvastatin (F_(2,27)_ = 38.00, P < 0.0001) decreased brain parasite load in animals chronically infected with *T*. *gondii* ME49 strain (P<0.0001) in relation to infected animals treated with vehicle (Fig 3).

**Fig 4:**
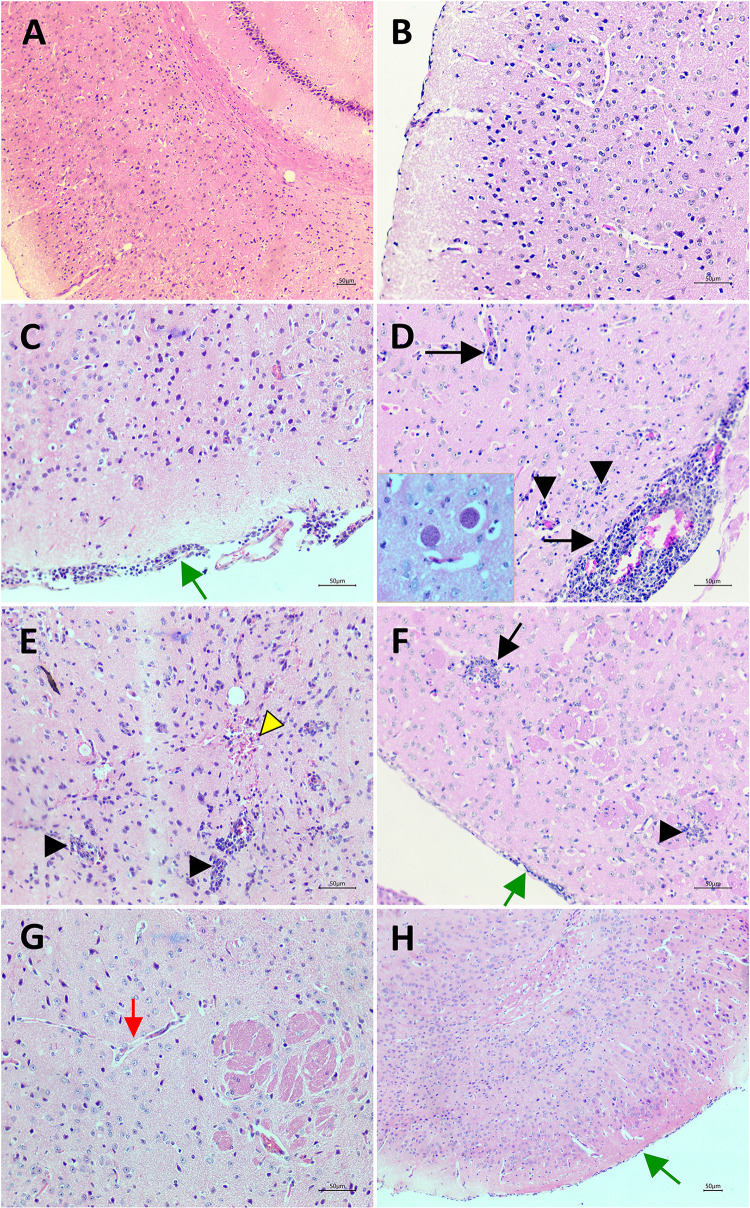
Histopatological analysis of brain tissue. (A—B) Normal structure of cortex from control group (non-infected). (C—E) Infected with *T*. *gondii* ME-49 strain, treated with vehicle group (infected control group). Meningitis (green arrow), perivascular cuffs (arrow), lymphocytes and microglial nodules (arrow head), hemorrhage (yellow arrow head). In detail, *Toxoplasma gondii* cysts. (F—G) Infected group treated with rosuvastatin 40mg/Kg. Discrete inflammatory cell infiltration and microglial proliferation with gliosis areas, indicating old tissue injury. Meninges show discrete inflammation (green arrow). Blood vessel without perivascular cuff (red arrow). (H) control group treated with rosuvastatin 40mg/Kg. Preserved nerve tissue and meninges (green arrow). (Scale bar = 50μm).

It was observed in the HE stained brain sections from experimentally chronic infected mice showed histopathological changes and inflammation signs, including meningoencephalitis, perivascular cuffs of lymphocytes, microglial nodules and tissue lesions with gliosis (Fig 4C–4E). Additionally, vascular congestion, hemorrhage and *T*. *gondii* cysts were observed in the brain (Fig 4C–4E). However, analysis of rosuvastatin treated infected group demonstrated the alterations such meningitis, perivascular infiltrate and inflammatory focus were attenuated and there were proliferation of glial cells and consolidated gliosis areas, indicating tissue repair to previous damage (Fig 4F and 4G). The control groups, vehicle (Fig 4A and 4B) or rosuvastatin treated (Fig 4H) do not show evidence of tissue injury and inflammation.

## Discussion

This study is the first to investigate the effect of rosuvastatin treatment in anxiogenic-like effect and memory impairment evoked by infection with *T*. *gondii* ME-49 strain. Rosuvastatin is a drug of the statin class, widely used to reduce serum cholesterol levels, as they are effective and well tolerated, even with prolonged use. Statins are 3-hydroxy-3-methylglutaryl-coenzyme A (HMG-CoA) reductase inhibitors in humans, but studies have demonstrated that they can affect parasite replication in vitro [24], possibly through blocking the isoprenoids synthesis in T. gondii, through the 1-deoxy-D-xylulose-5-phosphate (DOXP) pathway, which are essential for parasite metabolism [22, 23].

Our results indicated that rosuvastatin reversed both the anxiogenic-like effect promoted by *T*. *gondii* infection in the OF test and the impairment of short- and long-term memory in the NOR test. Results reported here also revealed that rosuvastatin decreased the parasite load, inflammation and damage in the brain, which might be involved in effects identified in the behavioral tests.

Behavioral analysis in the OF indicated that ME-49 infection decreased time spent in the center of the apparatus, which indicates an anxiogenic-like effect. Our findings are in line with previous evidence that *T*. *gondii* infection increased anxiety in the OF [37–40]. However, reports of the impact of *T*. *gondii* in anxiety are not consistent [31, 41, 42]. In fact, in addition to increase, decrease and none effect in anxiety-like behaviors have also been reported in several animal models of anxiety (e.g., OF, elevated plus-maze and social interaction test) in *T*. *gondii-*infected rodents [42]. It has been proposed that inconsistencies in behavioral changes might be related to differences in *T*. *gondii* strains, host species and sexes and methodologies to measure behavior [42]. Regarding methodology, it is worth mentioning that most studies evaluated anxiety in the OF [42]. Besides, decrease in center time in the present study was not followed by changes in either general locomotion or peripheral explorations, which reinforce the idea that change in center exploration is related to anxiety. Importantly, present study provides the first evidence that treatment with rosuvastatin inhibits the anxiogenic-like effect in the OF evoked by infection with *T*. *gondii* ME-49 strain. Such results are encouraging in the scope of investigations of treatments for chronic toxoplasmosis.

We also observed that *T*. *gondii* ME-49 strain infection caused impairment of both short- and long-term memory in the NOR test. Studies with this same strain also observed memory impairment [9, 43], effect that has also been reported following infection with other strains [9, 44]. The memory impairment provides further evidence that *T*. *gondii* infection affects the CNS. In addition to animal studies, there is evidence that CNS alterations in human chronic toxoplasmosis can be associated with cognitive changes. For instance, clinical studies observed association between chronic seropositivity for *T*. *gondii* and poor scholastic performance in the mathematics subtests [45–47]. Besides, children congenitally infected showed, after 10 years, learning difficulties and delay in school development [46]. The infection is believed to act directly and indirectly on the CNS, being related to psychiatric disorders such as schizophrenia, bipolar disorder and more subtle personality changes [48–51]. These results reveal the importance of understanding the mechanisms involved in behavioral alterations and memory impairment during *T*. *gondii* infection and, thus, developing new therapeutic targets. In this sense, the inhibition of memory impairment, in addition to the reversal of the anxiogenic effect identified in the present study in animals treated with rosuvastatin, indicates that statins can be an effective approach in the treatment of the various neurological and psychiatric manifestations related to *T*. *gondii* infection.

Our results showed that treatment with rosuvastatin reduced the parasitic burden of *T*. *gondii*, inflammation signs and histopathological changes in brain tissue. In the infected control group alterations such meningitis, inflammatory cell infiltration and perivascular cuffs were observed even after 71 days post infection, a similar result to a previous study from our research group that showed persistent inflammatory patterns in the chronic phase [26]. However, these pathological alterations were attenuated in the rosuvastatin treated infected group, which demonstrated more intense glial cell proliferation and gliosis area, indicating tissue repair. Other studies also demonstrated that *T*. *gondii* ME49-infected mice exhibit sustained cerebral inflammation even during chronic phase of disease [11, 26] and showing that rosuvastatin reduces brain inflammation and cerebral parasitic load in the chronic phase of infection with the same dose used in the present study (i.e., 40mg/kg) [26]. Behavioral changes have been shown to be related to cyst burden and brain inflammatory response in mice [11]. In fact, Xiao (2020) [11] demonstrated that plasmatic concentration of pro-inflammatory cytokines reflect the cyst burden in the CNS, and high levels of pro-inflammatory cytokines are positively correlated with behavioral changes in *T*. *gondii* infected mice. Accordingly, behavioral changes were reversed by reduction in neuroinflammation [52]. Study from Ihara et al. [34] demonstrated an association between fear memory impairment in T. gondii infected mice with inflammation and lesions in cerebral areas involved in the behavioral changes. In addition, these authors also found a cortical dysfunction resulting from alterations in dopamine metabolites levels in the cortex of the infected animals, indicating a possible relationship between altered neurotransmitter levels and behavioral changes following *T*. *gondii* infection. Furthermore, treatment with a combination of sulfamethoxazole-trimethoprim and resveratrol prevented behavioral changes in *T*. *gondii*-infected mice by reducing brain cyst number, which was followed by reduction in tissue lesions and oxidative levels [44]. The treatment of *T*. *gondii*-infected mice partly rescued the behavioral changes associated with *T*. *gondii* infection, suggesting that the degree of brain inflammation affects these behavioral changes [44]. Therefore, these findings support the idea that decreased brain lesion and inflammation identified in the present study in *T*.*gondii*-infected mice treated with rosuvastatin is possibly related to effects of this pharmacological treatment in anxiety and memory responses.

Results reported in the present study reinforce the importance of research on drugs that can act in the chronic phase of toxoplasmosis. In this sense, our findings provide evidence of the ability of rosuvastatin treatment to reverse memory impairment and symptoms related to anxiety, opening possibilities for further investigations on the efficacy of this drug in treatment of other neurological and psychiatric disorders related to toxoplasmosis. Tests of drugs already available on the market, such as rosuvastatin, can reduce costs and operations with studies on the mechanism of action, tolerance, mutagenesis, interactions, carcinogenicity, among others. Therefore, data of this study indicate rosuvastatin treatment as being a possible option of a new anti-*Toxoplasma* drug.

## Conclusion

In conclusion, *T*. *gondii* infection in mice increased anxiety-like behaviors and impaired short- and long-term memory. Rosuvastatin treatment reversed both these effects. Furthermore, rosuvastatin reduced the load cysts in the brain and attenuated signs of brain inflammation such as meningitis, perivascular cuffs, microglial proliferation, inflammatory cell infiltration and tissue damage. These findings suggest that the reduced parasite load can induce decreased brain inflammation, which in turn contributes to less damage in the brain, thus reducing behavioral changes. We believe that it is extremely important to conduct studies to continue our findings, such as studies using other *T*. *gondii* genotypes with greater pathogenicity, besides being the first study that investigated the effect of rosuvastatin treatment in anxiogenic-like effect and memory impairment evoked by infection with *T*. *gondii* ME-49 strain.

Additionally, this study confirm the presence of persistent inflammation in the chronic phase of experimental toxoplasmosis with possible association of this condition to behavioral changes. The inflammation and host immune response could be topics in study target for new therapeutic strategies in chronic toxoplasmosis.

## Supporting information

S1 Fig Control group 10x. (JPG)

S2 Fig Control group 20x. (JPG)

S3 Fig Infected control group—perivascular cuff 10x. (JPG)

S4 Fig Infected control group—perivascular cuff 20x. (JPG)

S5 Fig Infected control group—meninges 10x. (JPG)

S6 Fig Infected control group—meninges 20x. (JPG)

S7 Fig Infected control group—lesion 10x. (JPG)

S8 Fig Infected control group—lesion 20x. (JPG)

S9 Fig Infected control group—hemorrhage 10x. (JPG)

S10 Fig Infected control group—hemorrhage 20x. (JPG)

S11 Fig Infected control group—Cyst 20x. (JPG)

S12 Fig Infected control group—Cyst 40x. (JPG)

S13 Fig Infected control group Cysts 40x. (JPG)

S14 Fig Infected treated group—meninges 10x. (JPG)

S15 Fig Infected treated group—meninges 20x. (JPG)

S16 Fig Infected treated group 10x. (JPG)

S17 Fig Infected treated group 20x. (JPG)

S18 Fig Infected treated group - 20x. (JPG)

S19 Fig Infected treated group 40x. (JPG)

S20 Fig Non infected treated control group 10x. (JPG)

S21 Fig Non infected treated control group 20x. (JPG)

S1 Data (DOCX)

S1 File List and description of histopathological images. (DOCX)

## References

[1] Dubey JP. History of the discovery of the life cycle of Toxoplasma gondii. International Journal for Parasitology. 2009;39: 877–882. 10.1016/j.ijpara.2009.01.005 19630138

[2] Pittman KJ, Knoll LJ. Long-Term Relationships: the Complicated Interplay between the Host and the Developmental Stages of Toxoplasma gondii during Acute and Chronic Infections. Microbiology and Molecular Biology Reviews. 2015;79: 387–401. 10.1128/MMBR.00027-15 26335719 PMC4557073

[3] Wang Z-D, Liu H-H, Ma Z-X, Ma H-Y, Li Z-Y, Yang Z-B, et al. Toxoplasma gondii Infection in Immunocompromised Patients: A Systematic Review and Meta-Analysis. Frontiers in Microbiology. 2017;8: 389. 10.3389/fmicb.2017.00389 28337191 PMC5343064

[4] Duffy AR, O’Connell JR, Pavlovich M, Ryan KA, Lowry CA, Daue M, et al. Toxoplasma gondii Serointensity and Seropositivity: Heritability and Household-Related Associations in the Old Order Amish. International Journal of Environmental Research and Public Health. 2019;16: 3732. 10.3390/ijerph16193732 31623376 PMC6801611

[5] Fenta DA. Seroprevalence of Toxoplasma gondii among pregnant women attending antenatal clinics at Hawassa University comprehensive specialized and Yirgalem General Hospitals, in Southern Ethiopia. BMC Infectious Diseases. 2019;19: 1056. 10.1186/s12879-019-4694-8 31842783 PMC6916095

[6] Moncada PA, Montoya JG. Toxoplasmosis in the fetus and newborn: An update on prevalence, diagnosis and treatment. Expert Review of Anti-Infective Therapy. 2012;10: 815–828. 10.1586/eri.12.58 22943404

[7] Mendez OA, Koshy AA. Toxoplasma gondii: Entry, association, and physiological influence on the central nervous system. Gubbels M-J, editor. PLOS Pathogens. 2017;13: e1006351. 10.1371/journal.ppat.1006351 28727854 PMC5519211

[8] Bhadra R, Cobb DA, Weiss LM, Khan IA. Psychiatric Disorders in Toxoplasma Seropositive Patients—The CD8 Connection. Schizophrenia Bulletin. 2013;39: 485–489. 10.1093/schbul/sbt006 23427221 PMC3627775

[9] Bezerra ECM, dos Santos SV., dos Santos TCC, de Andrade HF, Meireles LR. Behavioral evaluation of BALB/c (Mus musculus) mice infected with genetically distinct strains of Toxoplasma gondii. Microbial Pathogenesis. 2019;126: 279–286. 10.1016/j.micpath.2018.11.021 30447421

[10] Bay-Richter C, Petersen E, Liebenberg N, Elfving B, Wegener G. Latent toxoplasmosis aggravates anxiety- and depressive-like behaviour and suggest a role of gene-environment interactions in the behavioural response to the parasite. Behavioural Brain Research. 2019;364: 133–139. 10.1016/j.bbr.2019.02.018 30768994

[11] Xiao J. Toxoplasma-induced Behavioral Changes: An Aspecific Consequence of Neuroinflammation. Trends in Parasitology. 2020;36: 317–318. 10.1016/j.pt.2020.01.005 32191847

[12] Burgdorf KS, Trabjerg BB, Pedersen MG, Nissen J, Banasik K, Pedersen OB, et al. Large-scale study of Toxoplasma and Cytomegalovirus shows an association between infection and serious psychiatric disorders. Brain, Behavior, and Immunity. 2019;79: 152–158. 10.1016/j.bbi.2019.01.026 30685531

[13] Chen X, Chen B, Hou X, Zheng C, Yang X, Ke J, et al. Association between Toxoplasma gondii infection and psychiatric disorders in Zhejiang, Southeastern China. Acta Tropica. 2019;192: 82–86. 10.1016/j.actatropica.2019.02.001 30731066

[14] Berdoy M, Webster JP, Macdonald DW. Fatal attraction in rats infected with Toxoplasma gondii. Proceedings of the Royal Society of London Series B: Biological Sciences. 2000;267: 1591–1594. 10.1098/rspb.2000.1182 11007336 PMC1690701

[15] Dunay IR, Gajurel K, Dhakal R, Liesenfeld O, Montoya JG. Treatment of Toxoplasmosis: Historical Perspective, Animal Models, and Current Clinical Practice. Clinical Microbiology Reviews. 2018;31. 10.1128/CMR.00057-17 30209035 PMC6148195

[16] Wilson C, Nizet V, Maldonado Y, Remington J, Krupp MA, Klein J. Infectious Diseases of the Fetus and Newborn. Infectious Diseases of the Fetus and Newborn Infant. Elsevier; 2011. 10.1016/C2009-0-50442-4

[17] Petersen E, Schmidt DR. Sulfadiazine and pyrimethamine in the postnatal treatment of congenital toxoplasmosis: what are the options? Expert Review of Anti-infective Therapy. 2003;1: 175–182. 10.1586/14787210.1.1.175 15482110

[18] Chew WK, Segarra I, Ambu S, Mak JW. Significant Reduction of Brain Cysts Caused by Toxoplasma gondii after Treatment with Spiramycin Coadministered with Metronidazole in a Mouse Model of Chronic Toxoplasmosis. Antimicrobial Agents and Chemotherapy. 2012;56: 1762–1768. 10.1128/AAC.05183-11 22271863 PMC3318357

[19] El-Zawawy LA, El-Said D, Mossallam SF, Ramadan HS, Younis SS. Preventive prospective of triclosan and triclosan-liposomal nanoparticles against experimental infection with a cystogenic ME49 strain of Toxoplasma gondii. Acta Tropica. 2015;141: 103–111. 10.1016/j.actatropica.2014.09.020 25305510

[20] Serranti D, Buonsenso D, Valentini P. Congenital toxoplasmosis treatment. European Review for Medical and Pharmacological Sciences. 2011;15: 193–198. Available: https://www.europeanreview.org/article/891 21434486

[21] Gay-Andrieu F, Fricker-Hidalgo H, Sickinger E, Espern A, Brenier-Pinchart M-P, Braun H-B, et al. Comparative evaluation of the ARCHITECT Toxo IgG, IgM, and IgG Avidity assays for anti-Toxoplasma antibodies detection in pregnant women sera. Diagnostic Microbiology and Infectious Disease. 2009;65: 279–287. 10.1016/j.diagmicrobio.2009.07.013 19822270

[22] Li Z-H, Li C, Szajnman SH, Rodriguez JB, Moreno SNJ. Synergistic Activity between Statins and Bisphosphonates against Acute Experimental Toxoplasmosis. Antimicrobial Agents and Chemotherapy. 2017;61. 10.1128/AAC.02628-16 28559264 PMC5527628

[23] Li Z-H, Ramakrishnan S, Striepen B, Moreno SNJ. Toxoplasma gondii Relies on Both Host and Parasite Isoprenoids and Can Be Rendered Sensitive to Atorvastatin. Blader IJ, editor. PLoS Pathogens. 2013;9: e1003665. 10.1371/journal.ppat.1003665 24146616 PMC3798403

[24] Sanfelice RA, da Silva SS, Bosqui LR, Miranda-Sapla MM, Barbosa BF, Silva RJ, et al. Pravastatin and simvastatin inhibit the adhesion, replication and proliferation of Toxoplasma gondii (RH strain) in HeLa cells. Acta Tropica. 2017;167: 208–215. 10.1016/j.actatropica.2016.12.006 28012901

[25] Sanfelice RA, Rodrigues Bosqui L, da Silva SS, Miranda-Sapla MM, Aparecido Panagio L, Navarro IT, et al. Proliferation of Toxoplasma gondii (RH strain) is inhibited by the combination of pravastatin and simvastatin with low concentrations of conventional drugs used in toxoplasmosis. Journal of Applied Biomedicine. 2018;16: 29–33. 10.1016/j.jab.2017.10.009

[26] Nishi L, Santana PL, Evangelista FF, Beletini LF, Souza AH, Mantelo FM, et al. Rosuvastatin reduced brain parasite burden in a chronic toxoplasmosis in vivo model and influenced the neuropathological pattern of ME-49 strain. Parasitology. 2020;147: 303–309. 10.1017/S0031182019001604 31727196 PMC10317618

[27] Coppens I. Targeting lipid biosynthesis and salvage in apicomplexan parasites for improved chemotherapies. Nature Reviews Microbiology. 2013;11: 823–835. 10.1038/nrmicro3139 24162026

[28] Neto-Ferreira R, Rocha VN, Souza-Mello V, Mandarim-de-Lacerda CA, de Carvalho JJ. Pleiotropic effects of rosuvastatin on the glucose metabolism and the subcutaneous and visceral adipose tissue behavior in C57Bl/6 mice. Diabetology & Metabolic Syndrome. 2013;5: 32. 10.1186/1758-5996-5-32 23816341 PMC3716873

[29] Prut L, Belzung C. The open field as a paradigm to measure the effects of drugs on anxiety-like behaviors: A review. European Journal of Pharmacology. 2003;463: 3–33. 10.1016/s0014-2999(03)01272-x 12600700

[30] Gould TD, Dao DT, Kovacsics CE. The open field test. Neuromethods. 2009. pp. 1–20. 10.1007/978-1-60761-303-9-1

[31] Seibenhener ML, Wooten MC. Use of the Open Field Maze to Measure Locomotor and Anxiety-like Behavior in Mice. Journal of Visualized Experiments. 2015;96: 52434. 10.3791/52434 25742564 PMC4354627

[32] Morais-Silva G, Fernandes-Santos J, Moreira-Silva D, Marin MT. Concomitant stress potentiates the preference for, and consumption of, ethanol induced by chronic pre-exposure to ethanol. Brazilian Journal of Medical and Biological Research. 2016;49: e5009. 10.1590/1414-431X20155009 26628398 PMC4681418

[33] Costa-Ferreira W, Morais-Silva G, Gomes-de-Souza L, Marin MT, Crestani CC. The AT1 Receptor Antagonist Losartan Does Not Affect Depressive-Like State and Memory Impairment Evoked by Chronic Stressors in Rats. Frontiers in Pharmacology. 2019;10: 705. 10.3389/fphar.2019.00705 31293424 PMC6598205

[34] Ihara F, Nishimura M, Muroi Y, Mahmoud ME, Yokoyama N, Nagamune K, et al. Toxoplasma gondii Infection in Mice Impairs Long-Term Fear Memory Consolidation through Dysfunction of the Cortex and Amygdala. Adams JH, editor. Infection and Immunity. 2016;84: 2861–2870. 10.1128/IAI.00217-16 27456832 PMC5038065

[35] Nanassy OZ, Haydock PV., Reed MW. Capture of genomic DNA on glass microscope slides. Analytical Biochemistry. 2007;365: 240–245. 10.1016/j.ab.2007.03.017 17442254 PMC1955222

[36] Jauregui LH, Higgins J, Zarlenga D, Dubey JP, Lunney JK. Development of a Real-Time PCR Assay for Detection of Toxoplasma gondii in Pig and Mouse Tissues. Journal of Clinical Microbiology. 2001;39: 2065–2071. 10.1128/JCM.39.6.2065-2071.2001 11376036 PMC88090

[37] Boillat M, Hammoudi P-M, Dogga SK, Pagès S, Goubran M, Rodriguez I, et al. Neuroinflammation-Associated Aspecific Manipulation of Mouse Predator Fear by Toxoplasma gondii. Cell Reports. 2020;30: 320–334.e6. 10.1016/j.celrep.2019.12.019 31940479 PMC6963786

[38] Hay J, Hutchison WM, Aitken PP, Graham DI. The effect of congenital and adult-acquired Toxoplasma infections on activity and responsiveness to novel stimulation in mice. Annals of Tropical Medicine & Parasitology. 1983;77: 483–495. 10.1080/00034983.1983.11811741 6660954

[39] Hay J, Aitken PP, Hair DM, Hutchison WM, Graham DI. The effect of congenital Toxoplasma infection on mouse activity and relative preference for exposed areas over a series of trials. Annals of Tropical Medicine & Parasitology. 1984;78: 611–618. 10.1080/00034983.1984.11811872 6532331

[40] Skallová A, Kodym P, Frynta D, Flegr J. The role of dopamine in Toxoplasma-induced behavioural alterations in mice: an ethological and ethopharmacological study. Parasitology. 2006;133: 525. 10.1017/S0031182006000886 16882355

[41] Webster JP, McConkey GA. Toxoplasma gondii-altered host behaviour: clues as to mechanism of action. Folia Parasitologica. 2010;57: 95–104. 10.14411/fp.2010.012 20608471

[42] Worth AR, Andrew Thompson RC, Lymbery AJ. Reevaluating the Evidence for Toxoplasma gondii-Induced Behavioural Changes in Rodents. Advances in Parasitology. 2014. pp. 109–142. 10.1016/B978-0-12-800182-0.00003-9 24928181

[43] Kannan G, Moldovan K, Xiao J-C, Yolken RH, Jones-Brando L, Pletnikov MV. Toxoplasma gondii strain-dependent effects on mouse behaviour. Folia Parasitologica. 2010;57: 151–155. 10.14411/fp.2010.019 20608478

[44] Bottari NB, Baldissera MD, Tonin AA, Rech VC, Alves CB, D’Avila F, et al. Synergistic effects of resveratrol (free and inclusion complex) and sulfamethoxazole-trimetropim treatment on pathology, oxidant/antioxidant status and behavior of mice infected with Toxoplasma gondii. Microbial Pathogenesis. 2016;95: 166–174. 10.1016/j.micpath.2016.04.002 27057672

[45] Ferreira EC, Marchioro AA, Guedes TA, Mota DCGA, Guilherme ALF, de Araujo SM. Association between seropositivity for Toxoplasma gondii, scholastic development of children and risk factors for T. gondii infection. Transactions of the Royal Society of Tropical Medicine and Hygiene. 2013;107: 390–396. 10.1093/trstmh/trt026 23598948

[46] Sanders AP, dos Santos T, Felipe CKK, Estevão ML, Cícero C, Evangelista F, et al. Ocular Lesions in Congenital Toxoplasmosis in Santa Isabel do Ivaí, Paraná, Brazil. Pediatric Infectious Disease Journal. 2017;36: 817–820. 10.1097/INF.0000000000001614 28640004

[47] Shwab EK, Saraf P, Zhu X-Q, Zhou D-H, McFerrin BM, Ajzenberg D, et al. Human impact on the diversity and virulence of the ubiquitous zoonotic parasite Toxoplasma gondii. Proceedings of the National Academy of Sciences. 2018;115: E6956–E6963. 10.1073/pnas.1722202115 29967142 PMC6055184

[48] Flegr J. Influence of latent Toxoplasma infection on human personality, physiology and morphology: pros and cons of the Toxoplasma-human model in studying the manipulation hypothesis. Journal of Experimental Biology. 2013;216: 127–133. 10.1242/jeb.073635 23225875

[49] Sutterland AL, Fond G, Kuin A, Koeter MWJ, Lutter R, van Gool T, et al. Beyond the association. Toxoplasma gondii in schizophrenia, bipolar disorder, and addiction: systematic review and meta-analysis. Acta Psychiatrica Scandinavica. 2015;132: 161–179. 10.1111/acps.12423 25877655

[50] Pearce BD, Kruszon-Moran D, Jones JL. The Relationship Between Toxoplasma Gondii Infection and Mood Disorders in the Third National Health and Nutrition Survey. Biological Psychiatry. 2012;72: 290–295. 10.1016/j.biopsych.2012.01.003 22325983 PMC4750371

[51] Yolken RH, Dickerson FB, Fuller-Torrey E. Toxoplasma and schizophrenia. Parasite Immunology. 2009;31: 706–715. 10.1111/j.1365-3024.2009.01131.x 19825110

[52] Martynowicz J, Augusto L, Wek RC, Boehm SL, Sullivan WJ. Guanabenz Reverses a Key Behavioral Change Caused by Latent Toxoplasmosis in Mice by Reducing Neuroinflammation. Boothroyd JC, editor. mBio. 2019;10: e00381–19. 10.1128/mBio.00381-19 31040237 PMC6495372

