# Supplementary figures and images for: Rosuvastatin revert memory impairment and anxiogenic-like effect in mice infected with the chronic ME-49 strain of Toxoplasma gondii
Source: PLoS One. 2021 Apr 15;16(4):e0250079. doi: 10.1371/journal.pone.0250079 (PMC8049280; doi:10.1371/journal.pone.0250079)

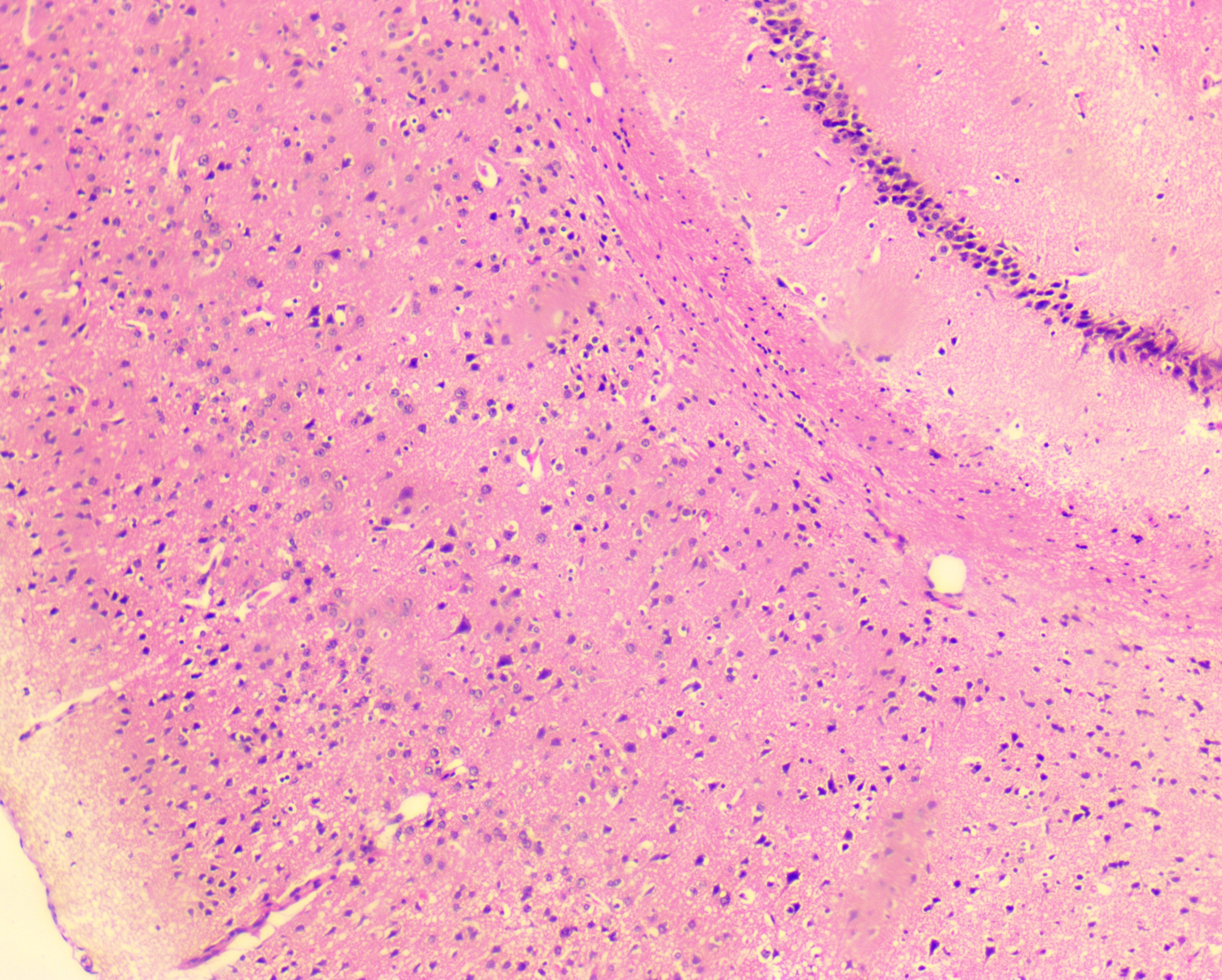

Supplement: S1 Fig — (JPG) [file pone.0250079.s001.jpg]

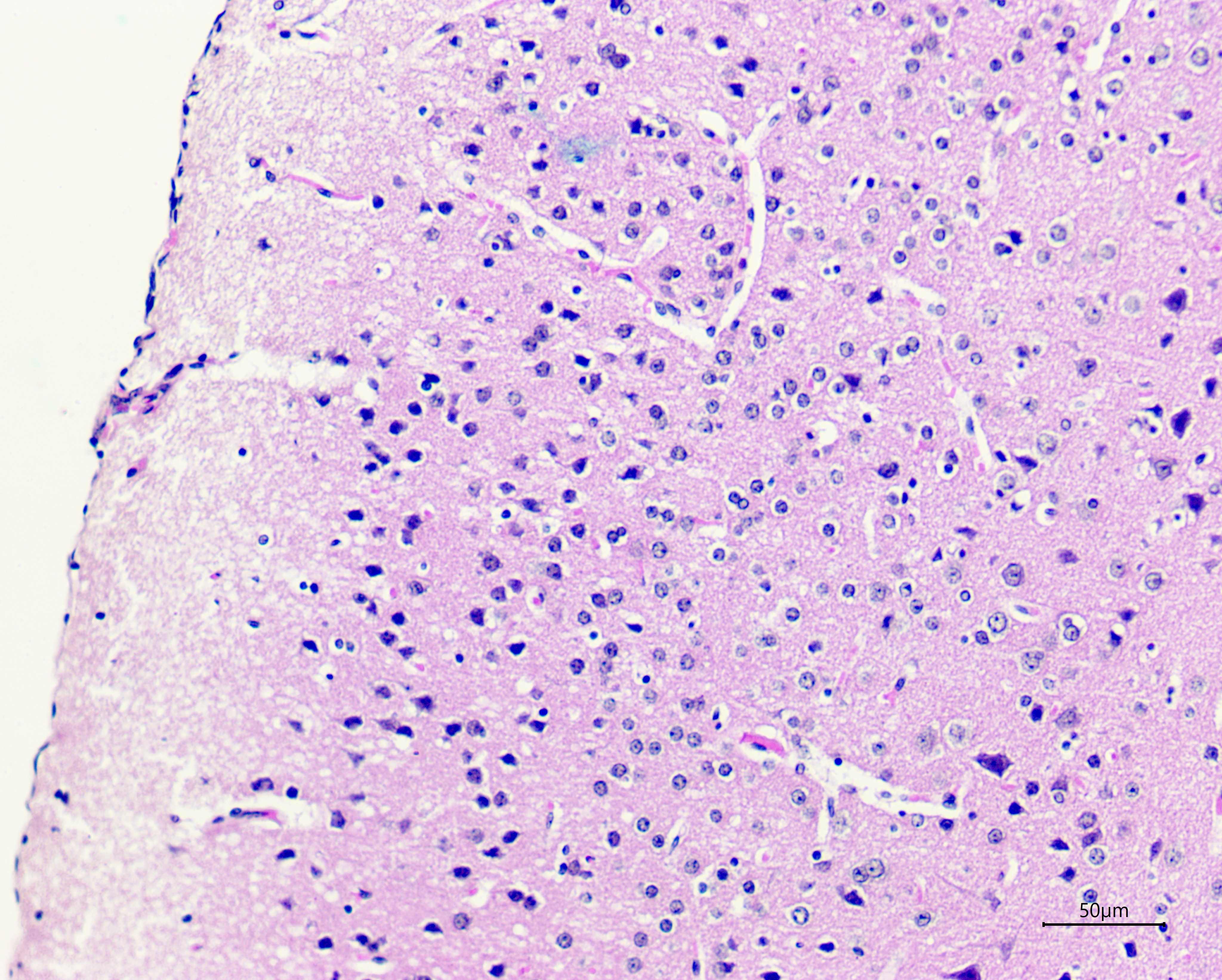

Supplement: S2 Fig — (JPG) [file pone.0250079.s002.jpg]

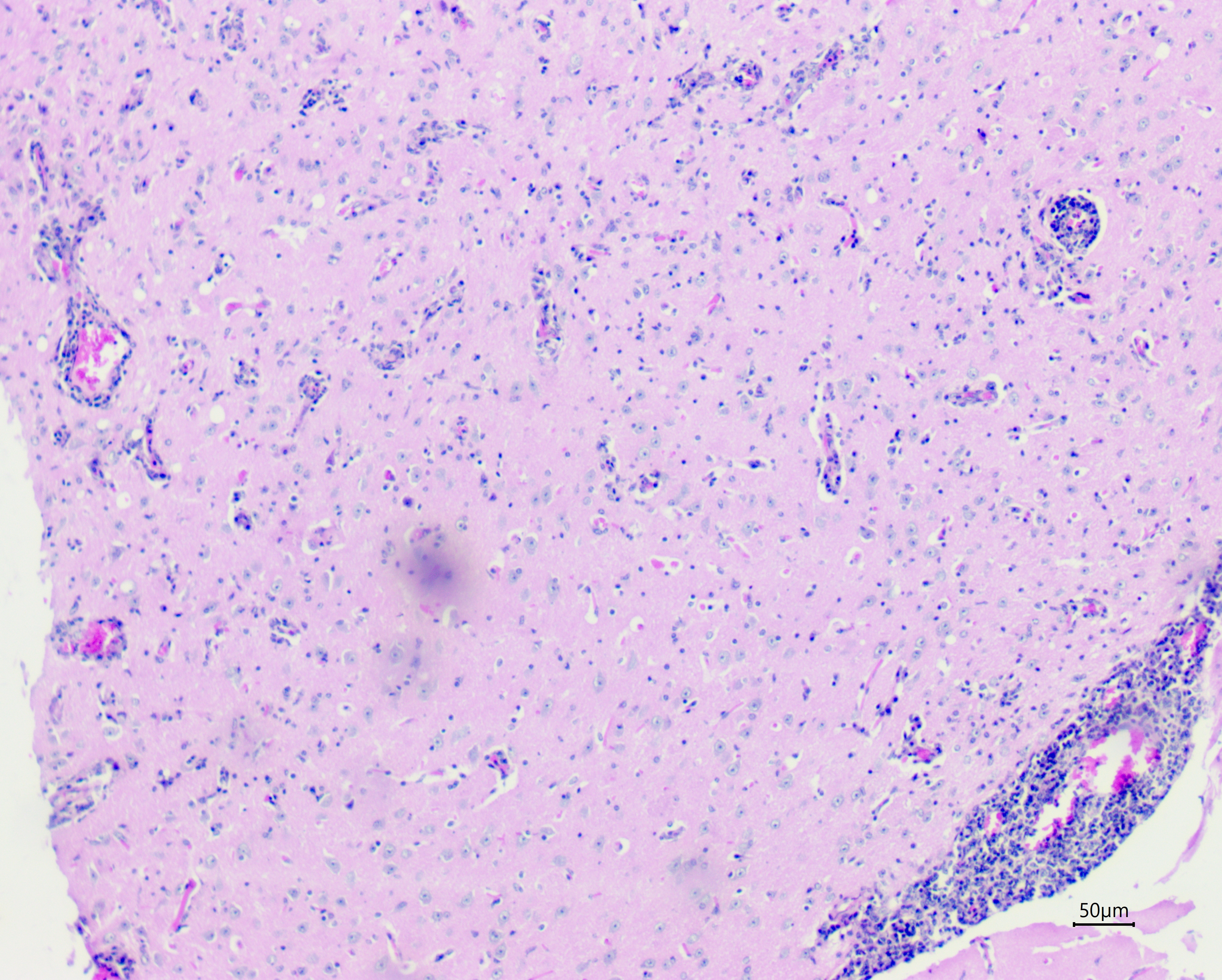

Supplement: S3 Fig — (JPG) [file pone.0250079.s003.jpg]

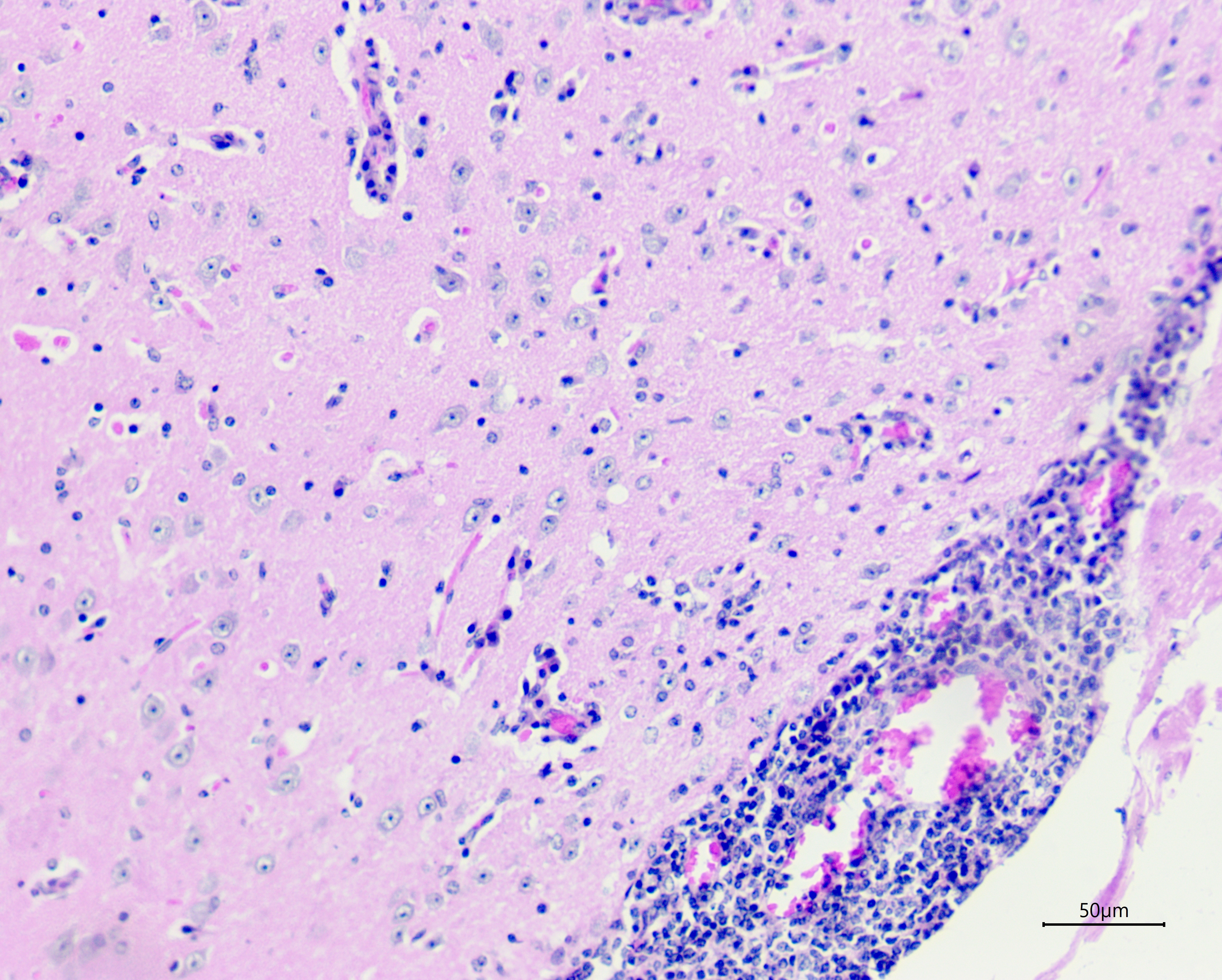

Supplement: S4 Fig — (JPG) [file pone.0250079.s004.jpg]

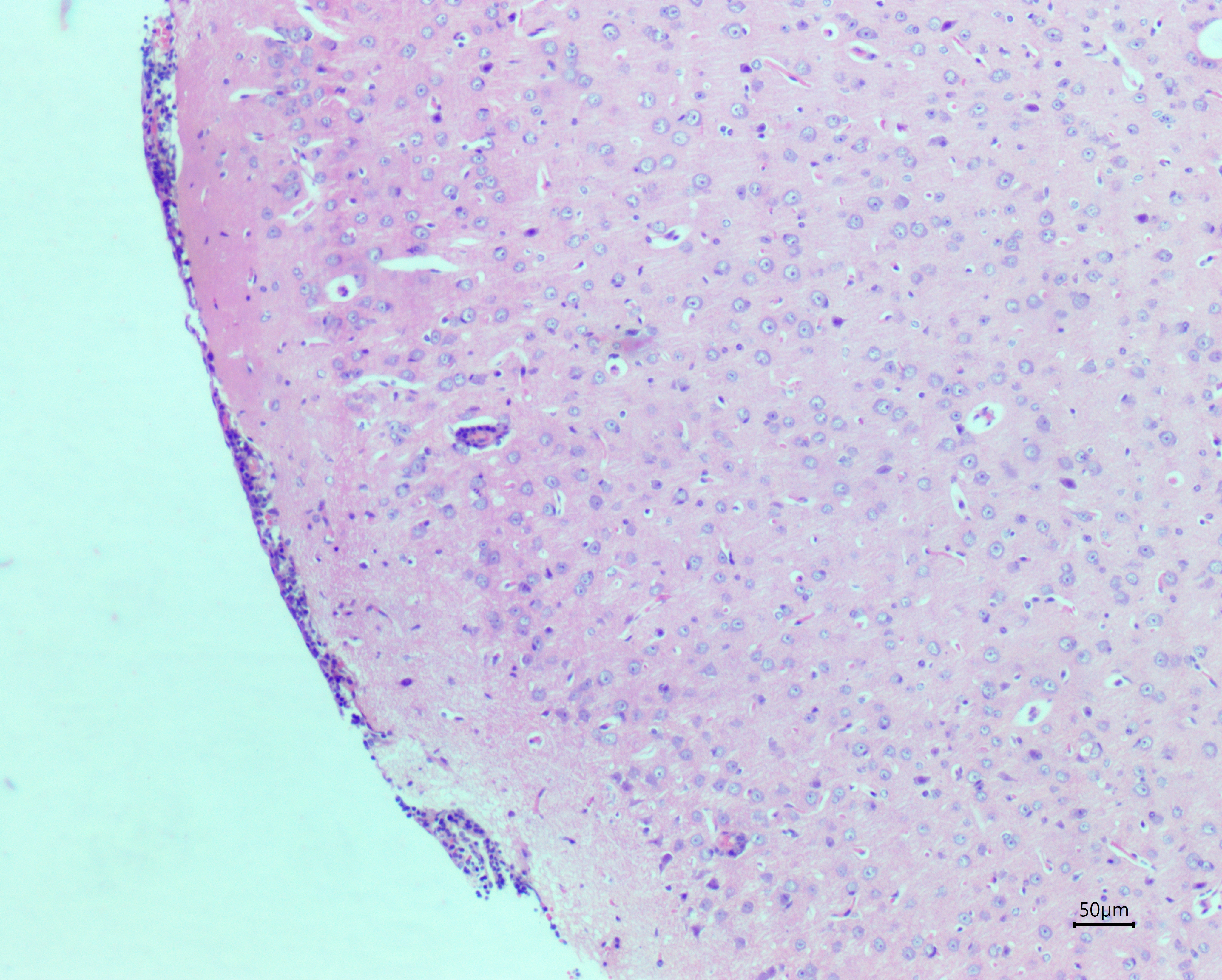

Supplement: S5 Fig — (JPG) [file pone.0250079.s005.jpg]

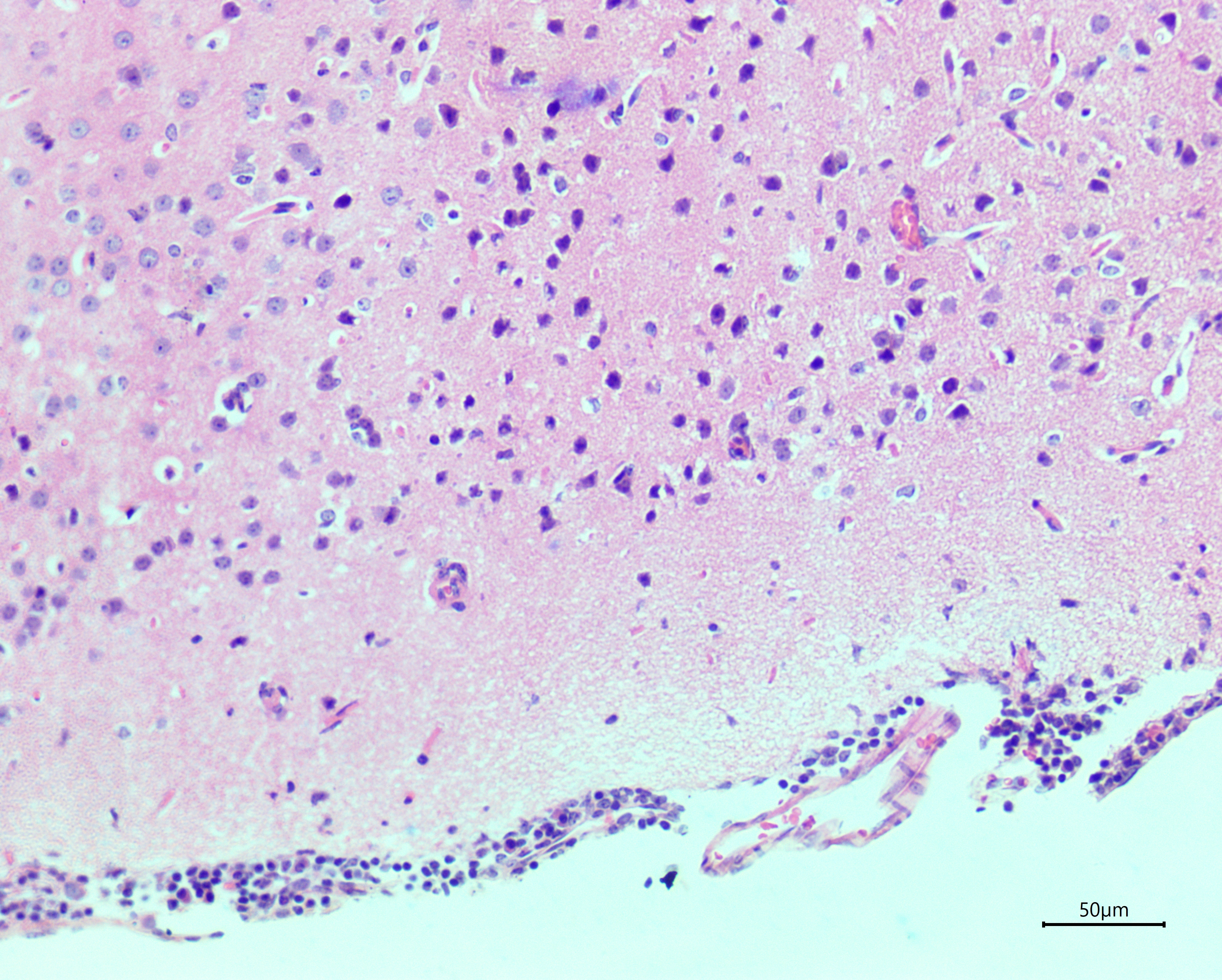

Supplement: S6 Fig — (JPG) [file pone.0250079.s006.jpg]

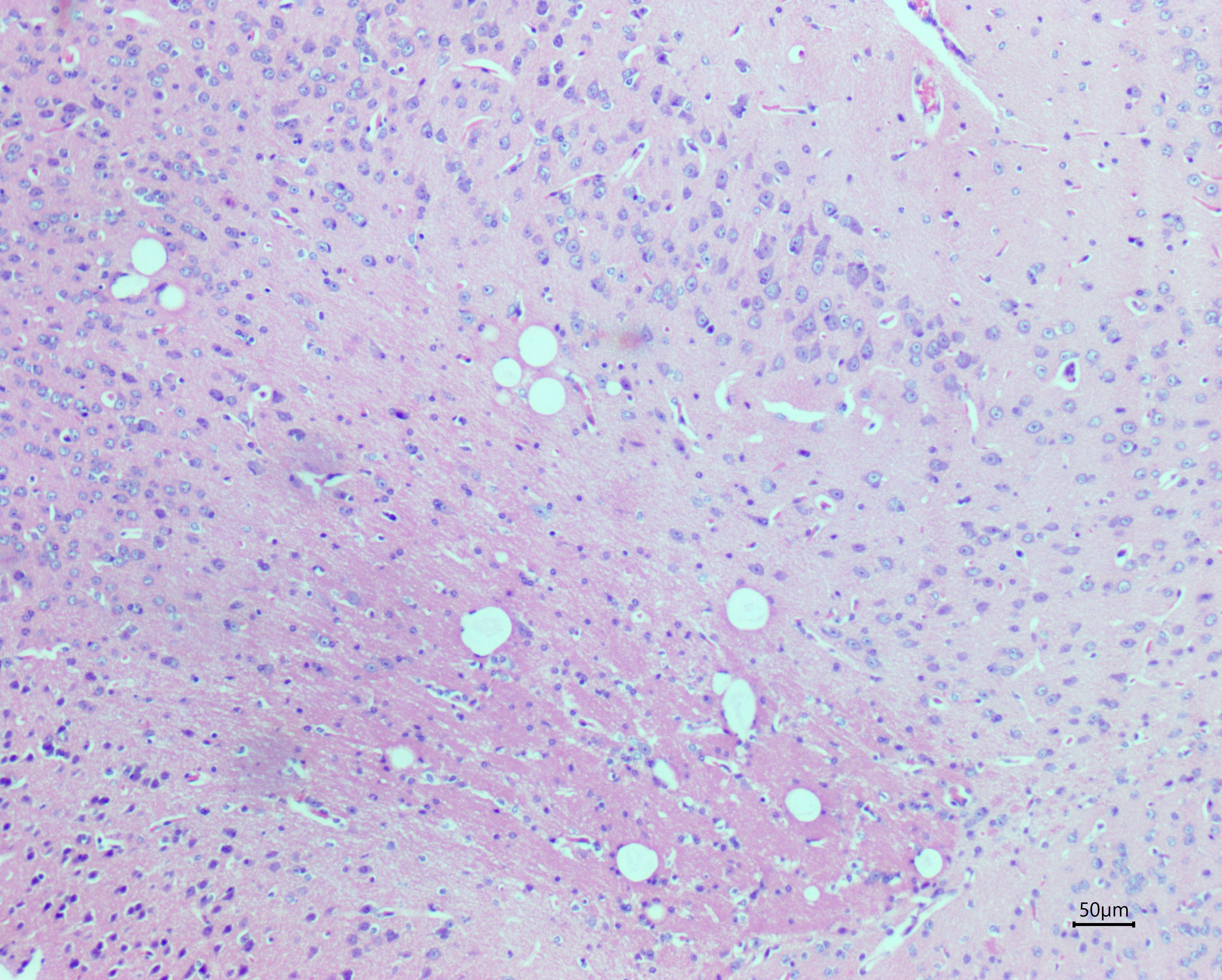

Supplement: S7 Fig — (JPG) [file pone.0250079.s007.jpg]

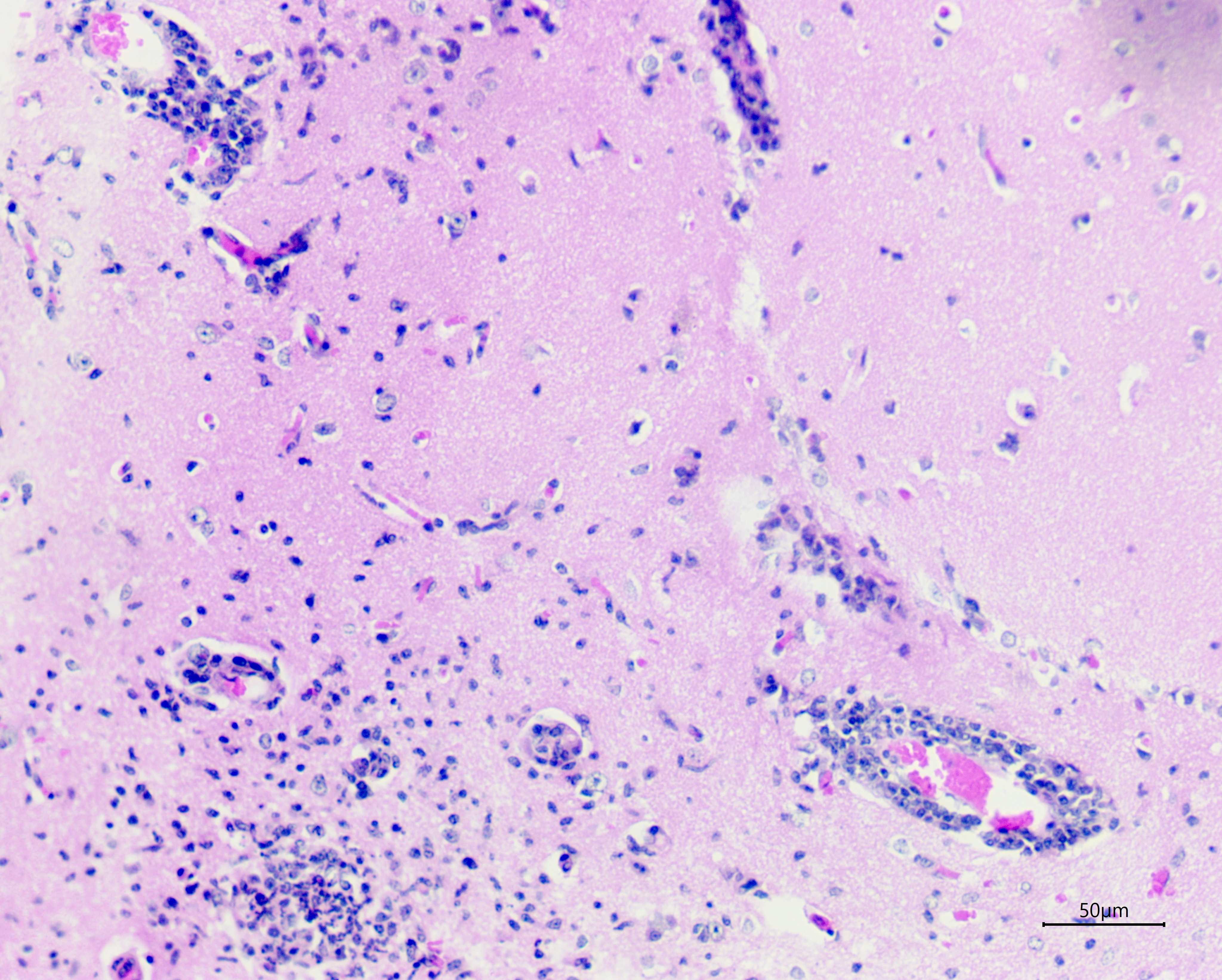

Supplement: S8 Fig — (JPG) [file pone.0250079.s008.jpg]

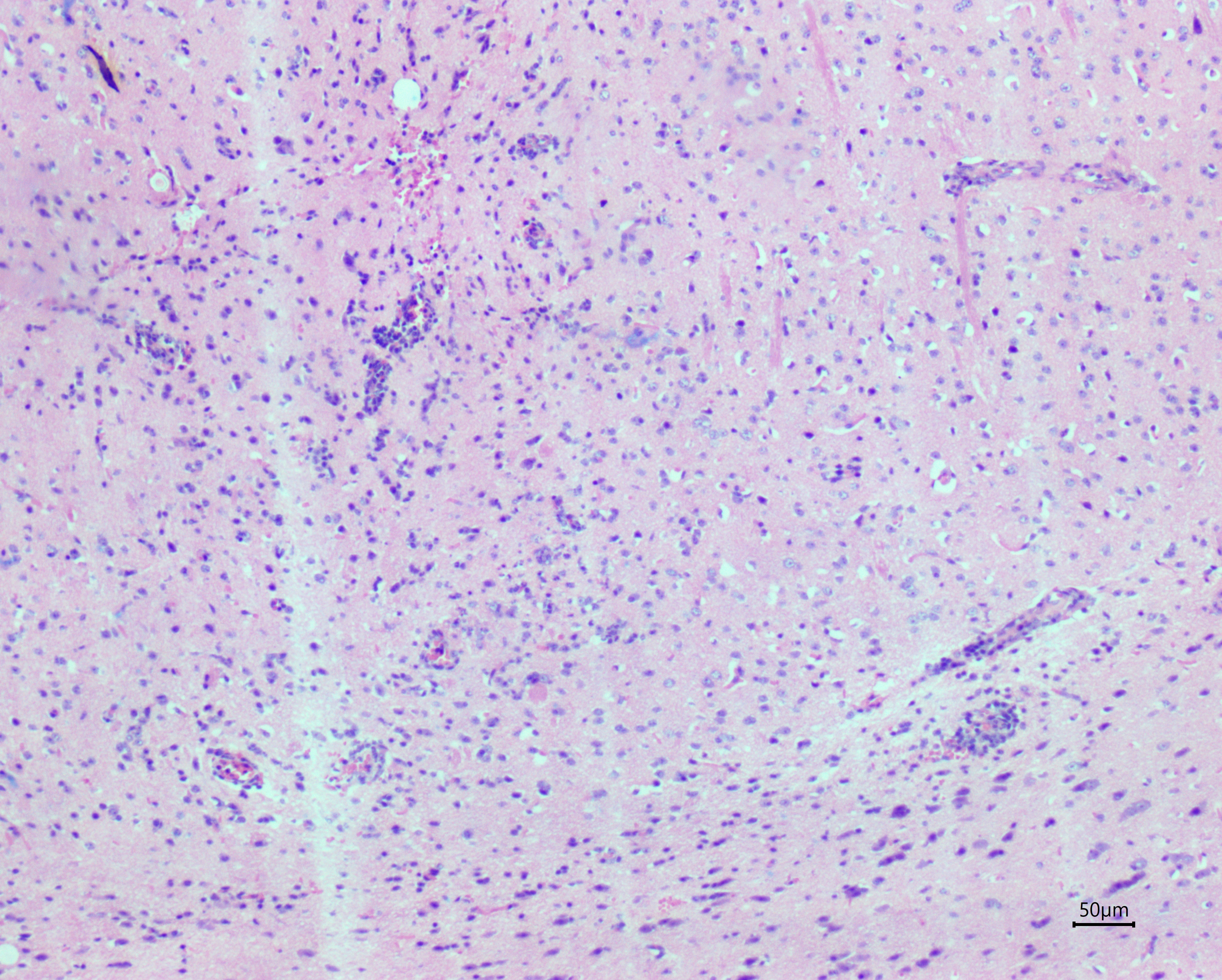

Supplement: S9 Fig — (JPG) [file pone.0250079.s009.jpg]

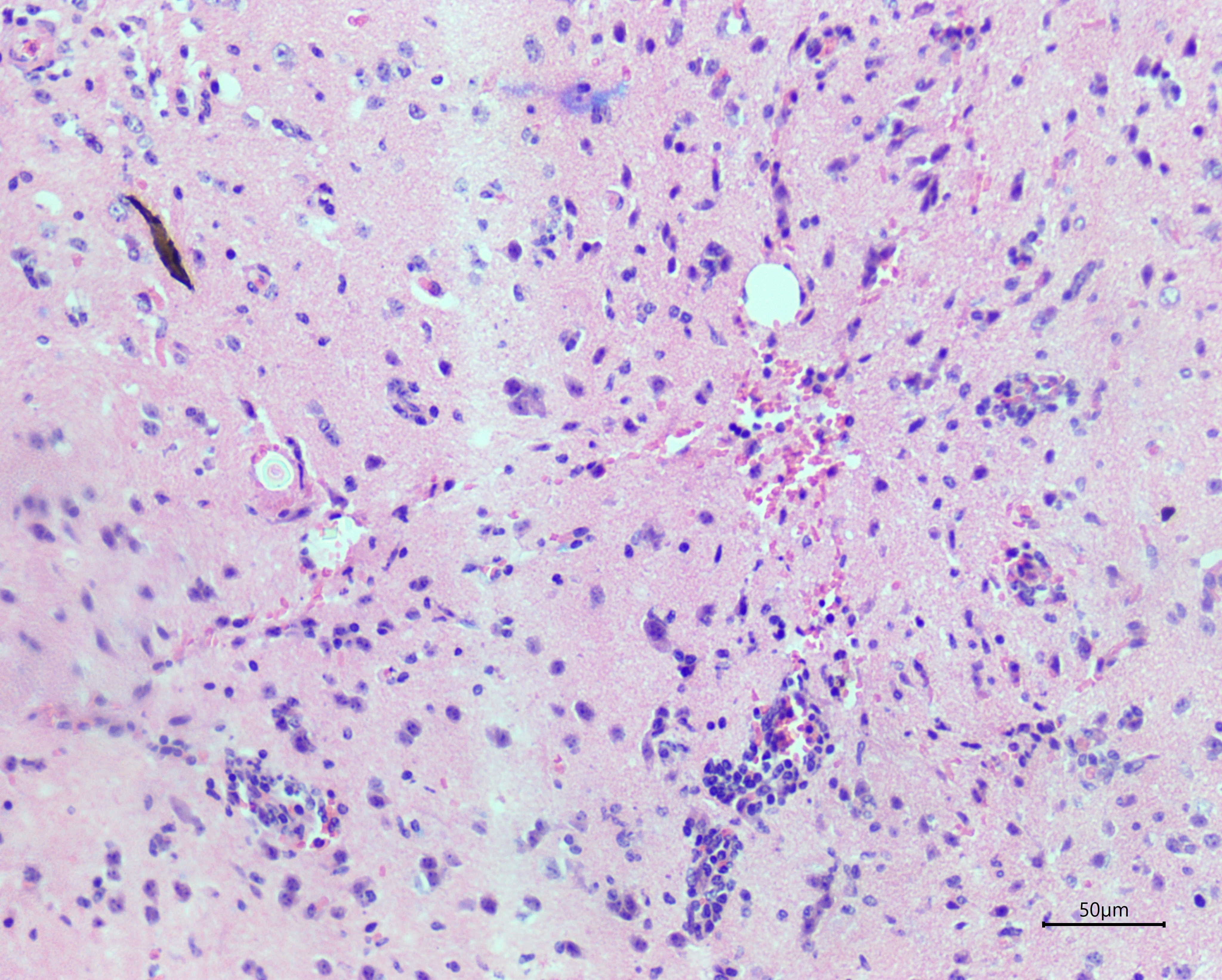

Supplement: S10 Fig — (JPG) [file pone.0250079.s010.jpg]

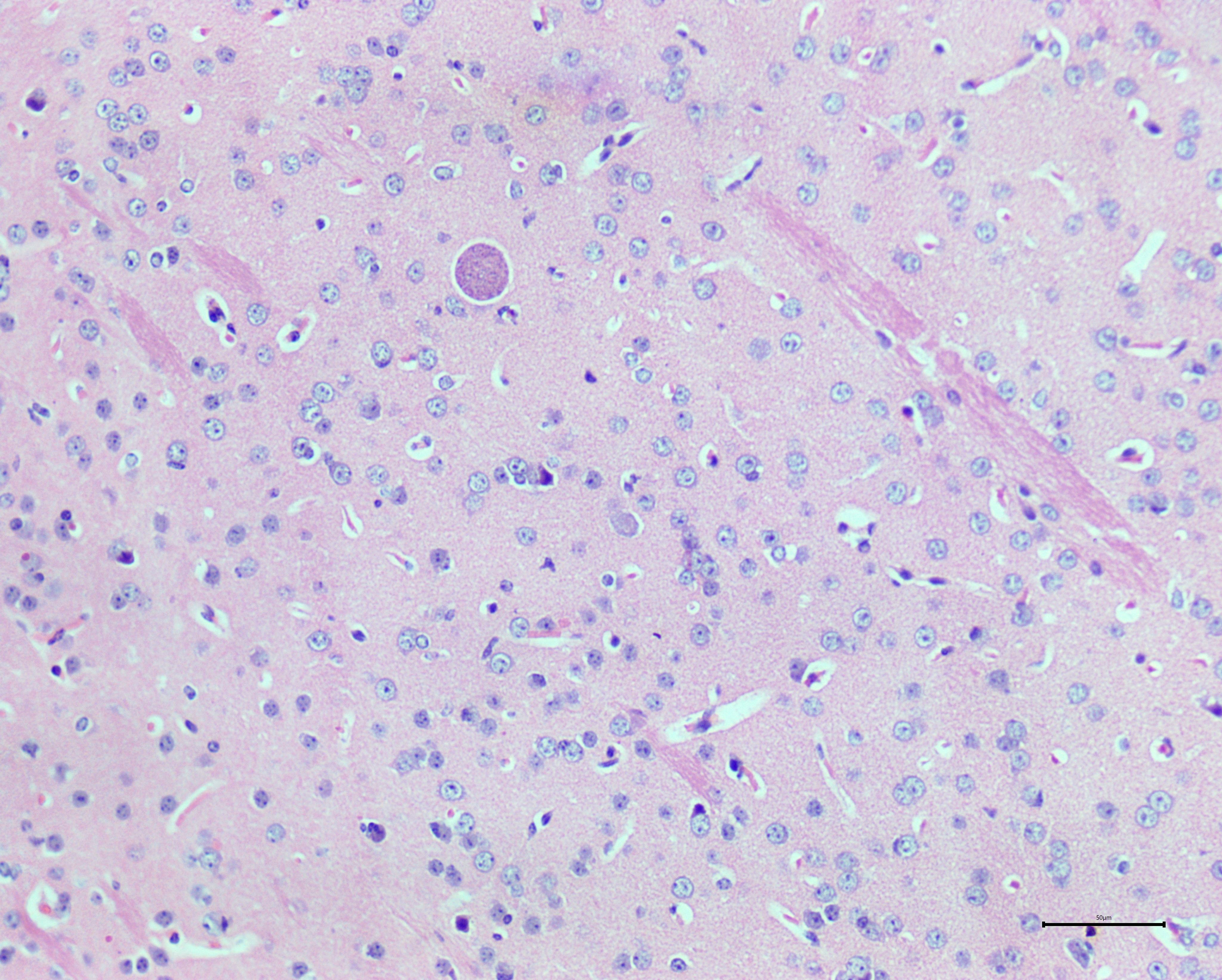

Supplement: S11 Fig — (JPG) [file pone.0250079.s011.jpg]

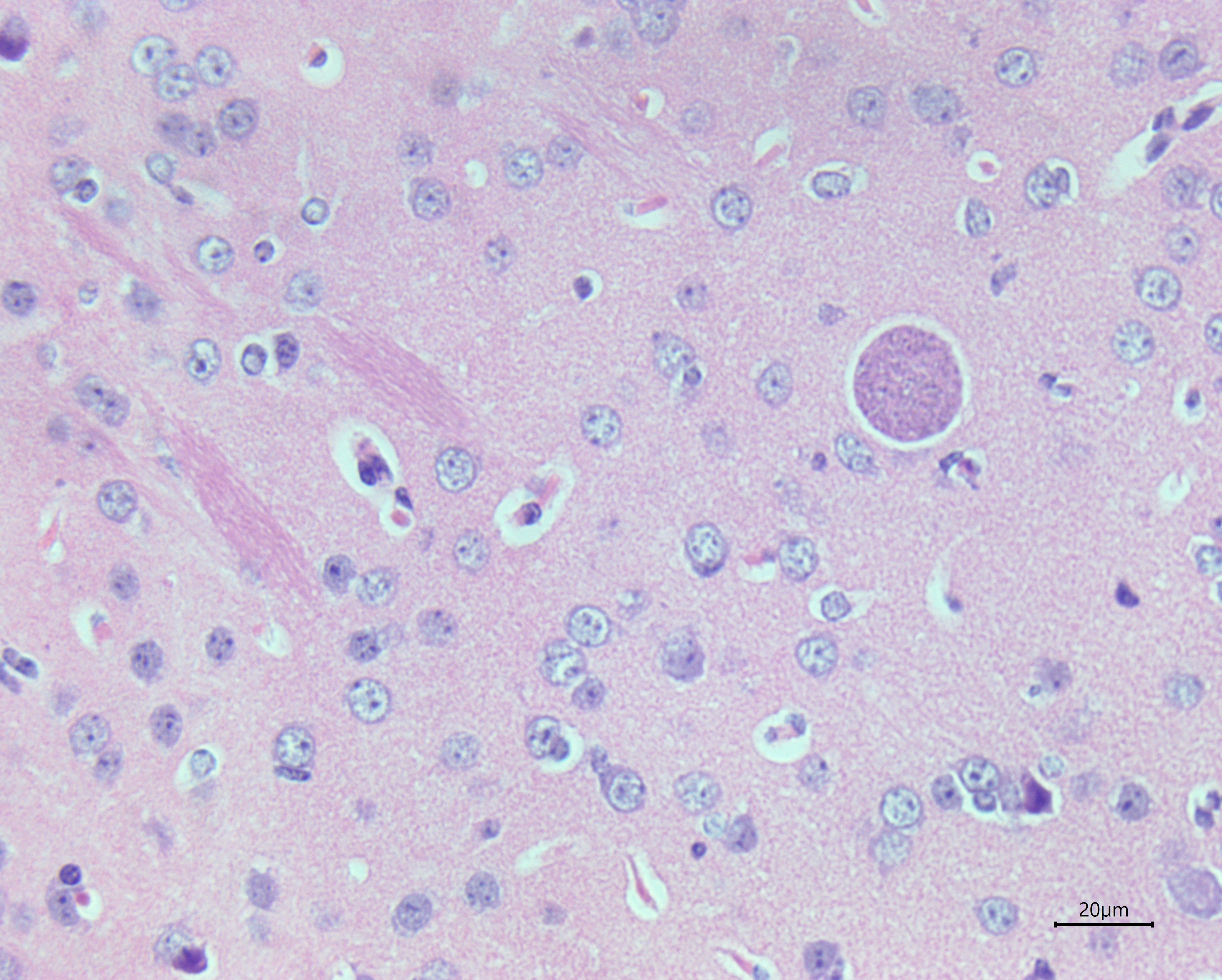

Supplement: S12 Fig — (JPG) [file pone.0250079.s012.jpg]

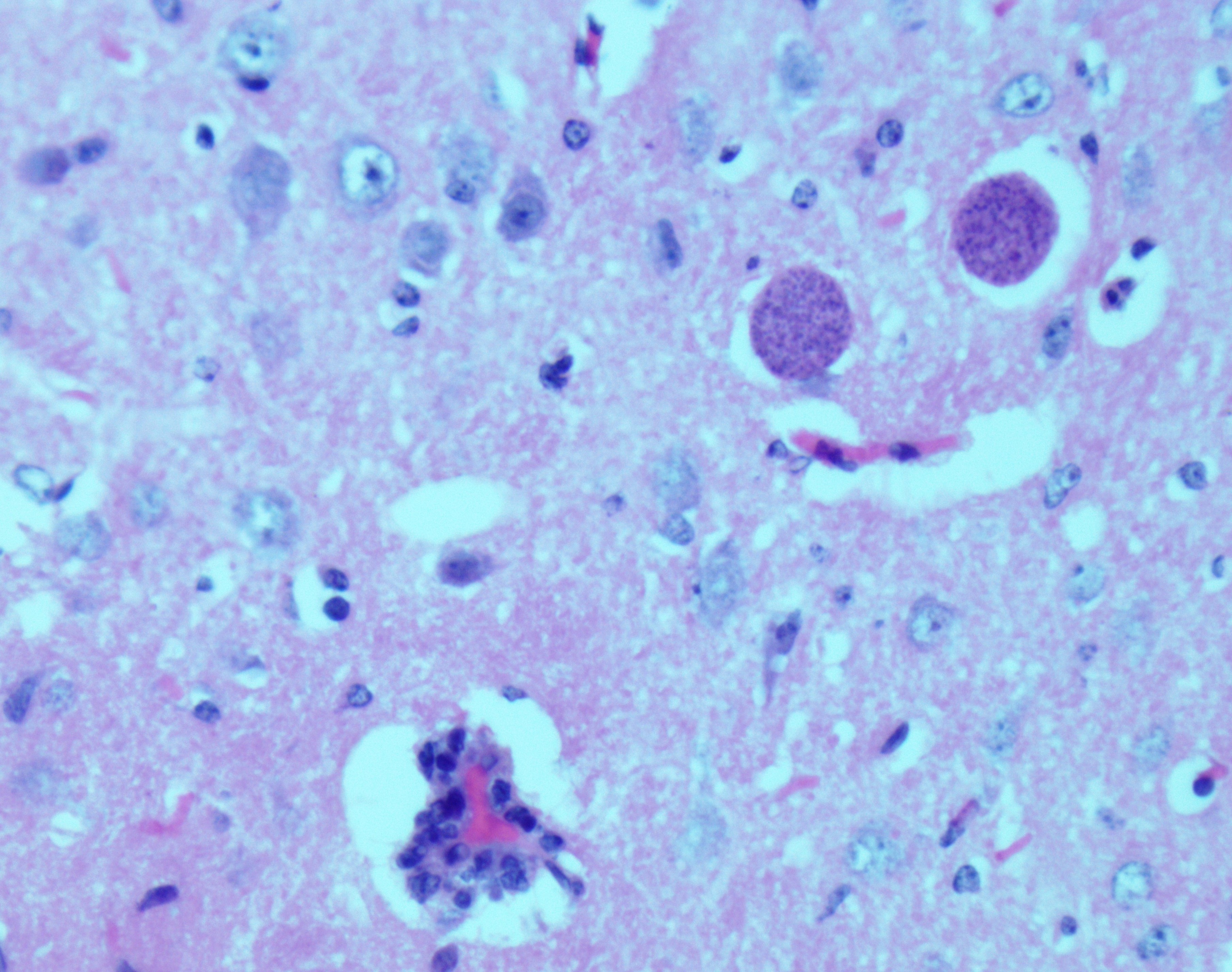

Supplement: S13 Fig — (JPG) [file pone.0250079.s013.jpg]

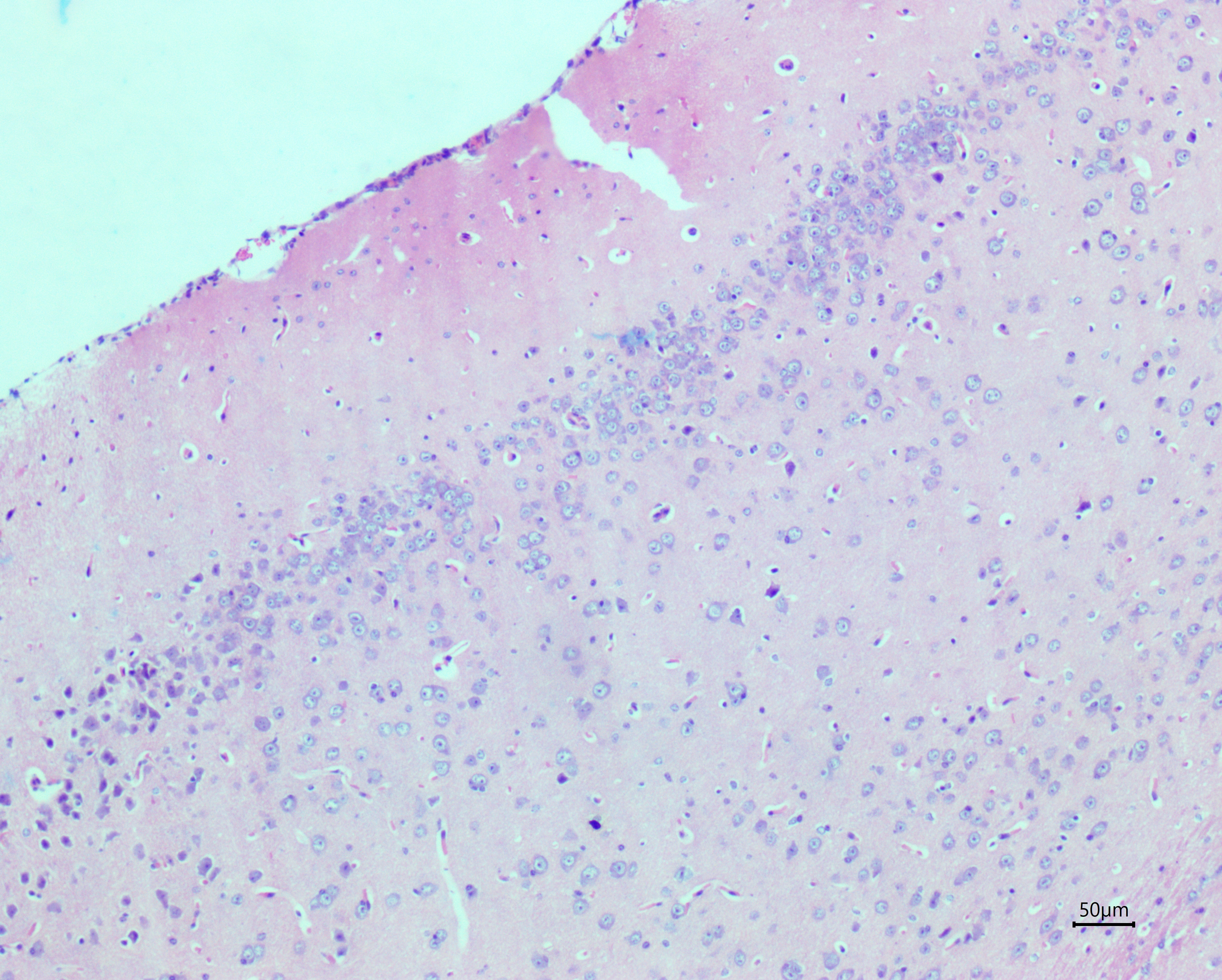

Supplement: S14 Fig — (JPG) [file pone.0250079.s014.jpg]

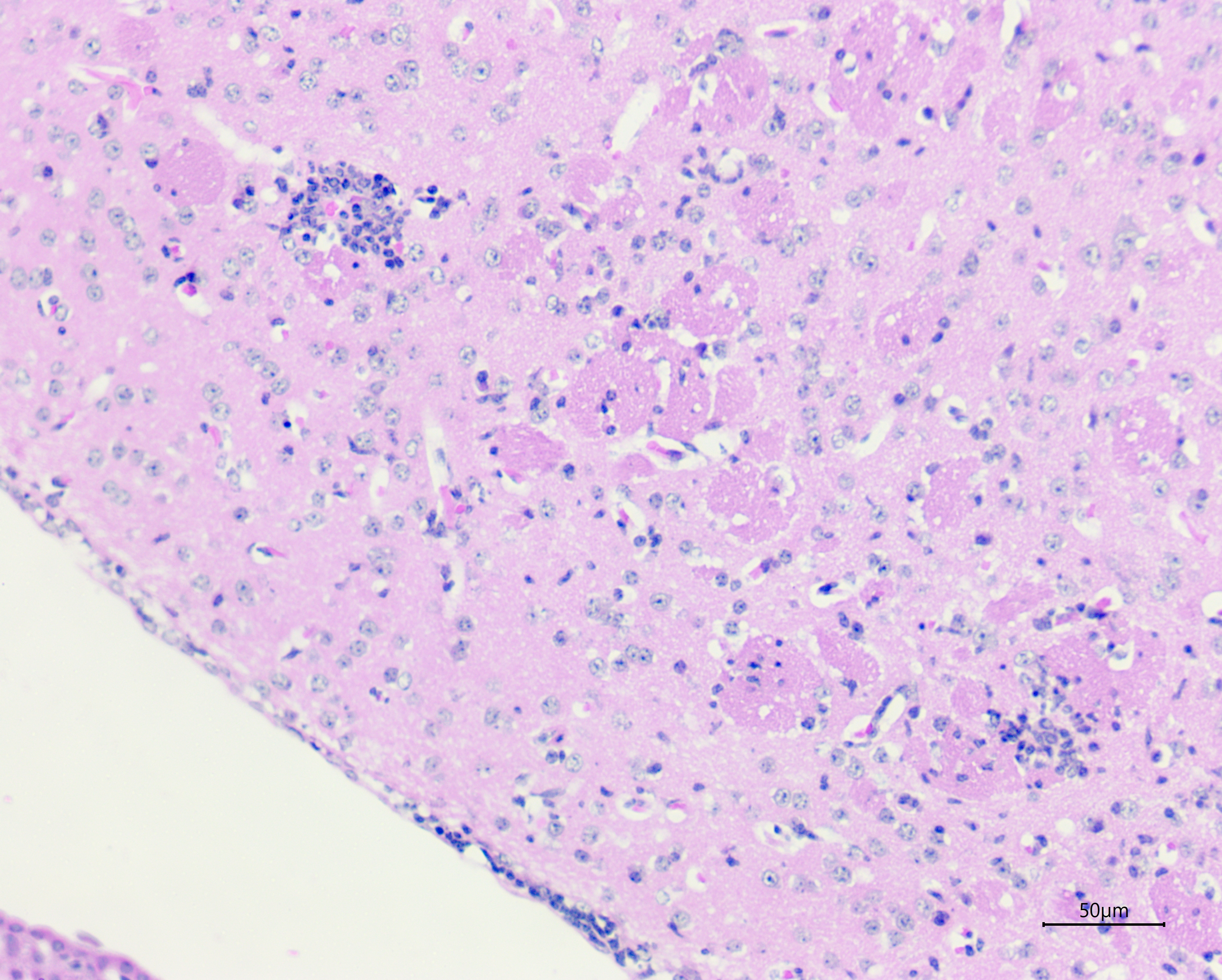

Supplement: S15 Fig — (JPG) [file pone.0250079.s015.jpg]

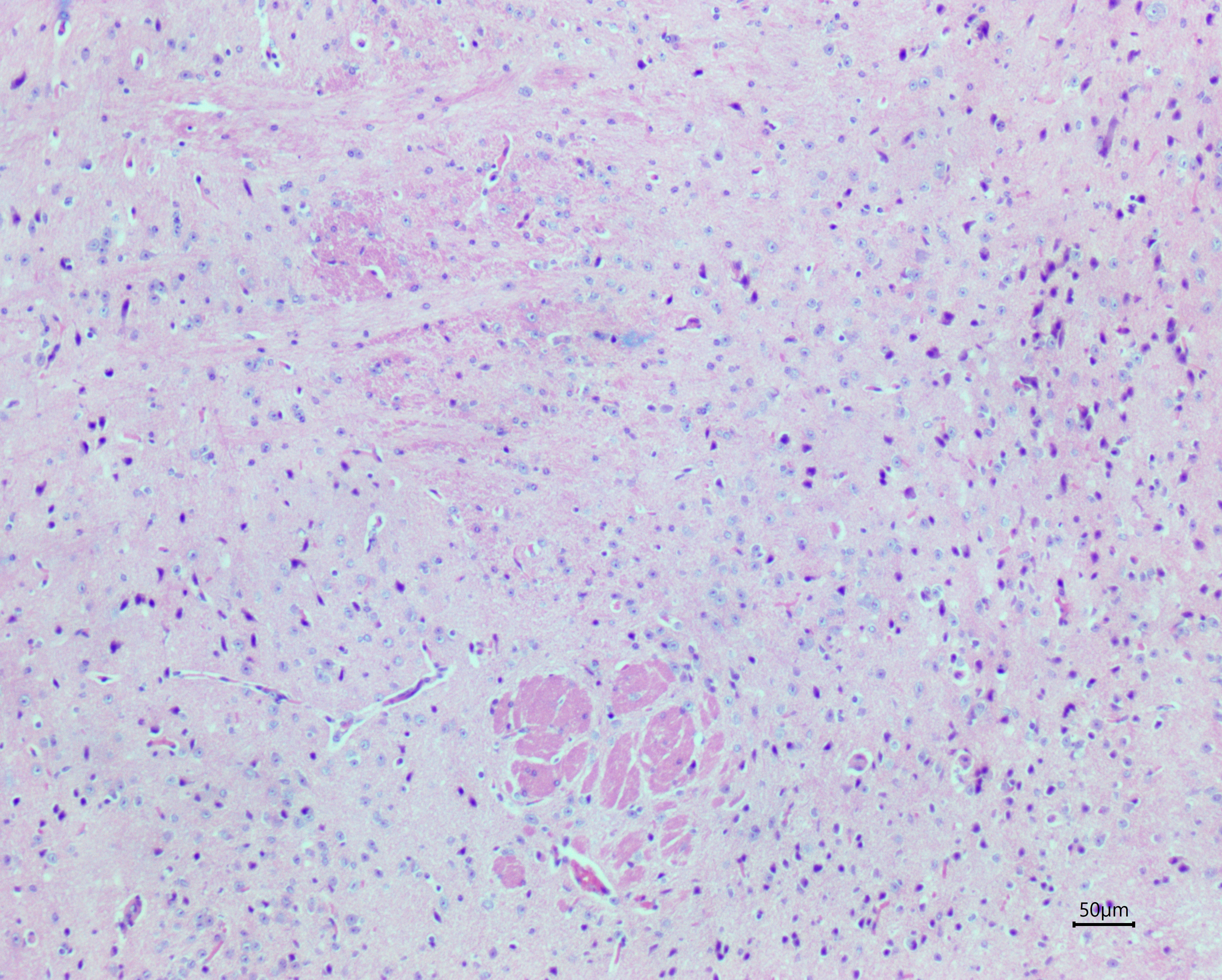

Supplement: S16 Fig — (JPG) [file pone.0250079.s016.jpg]

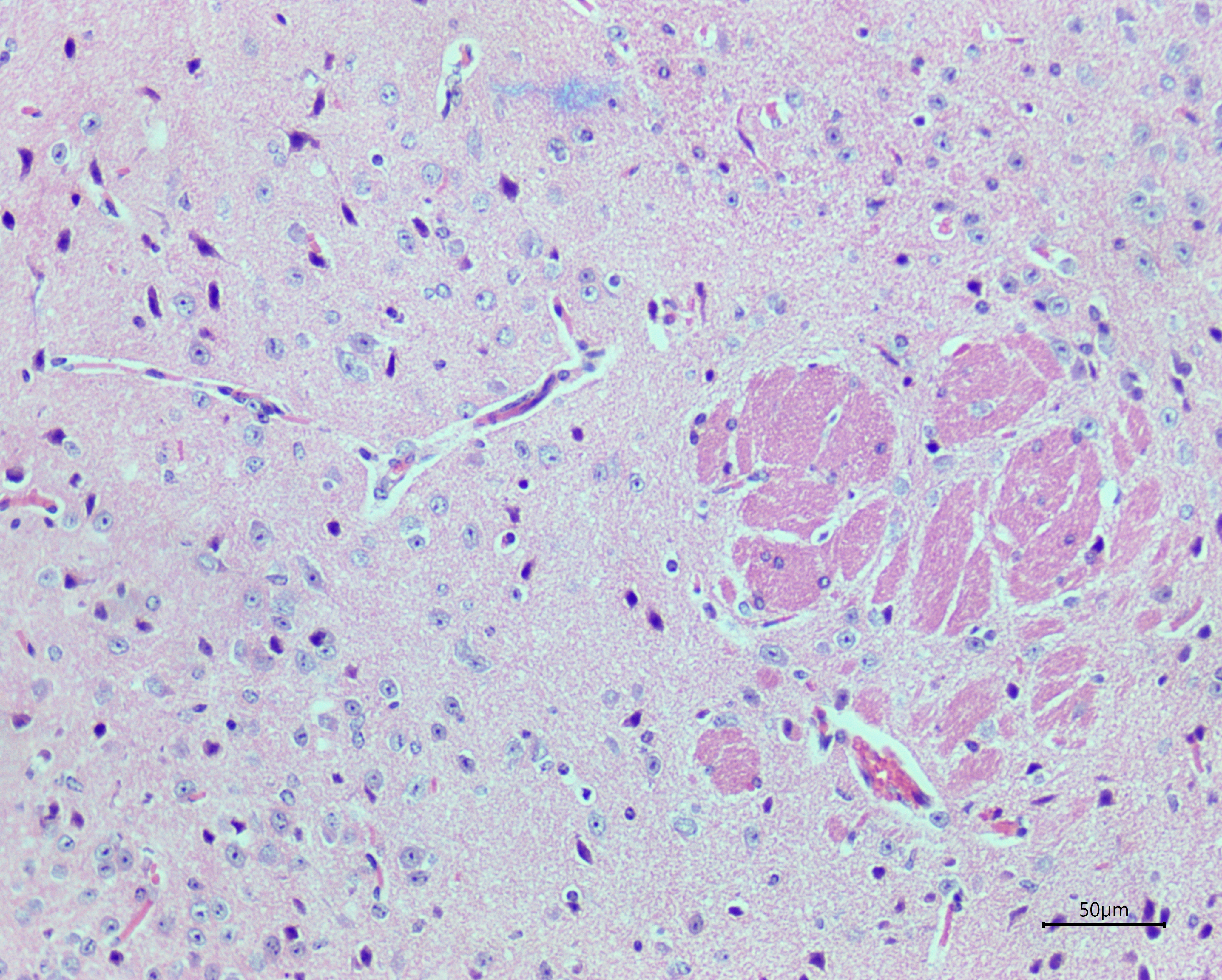

Supplement: S17 Fig — (JPG) [file pone.0250079.s017.jpg]

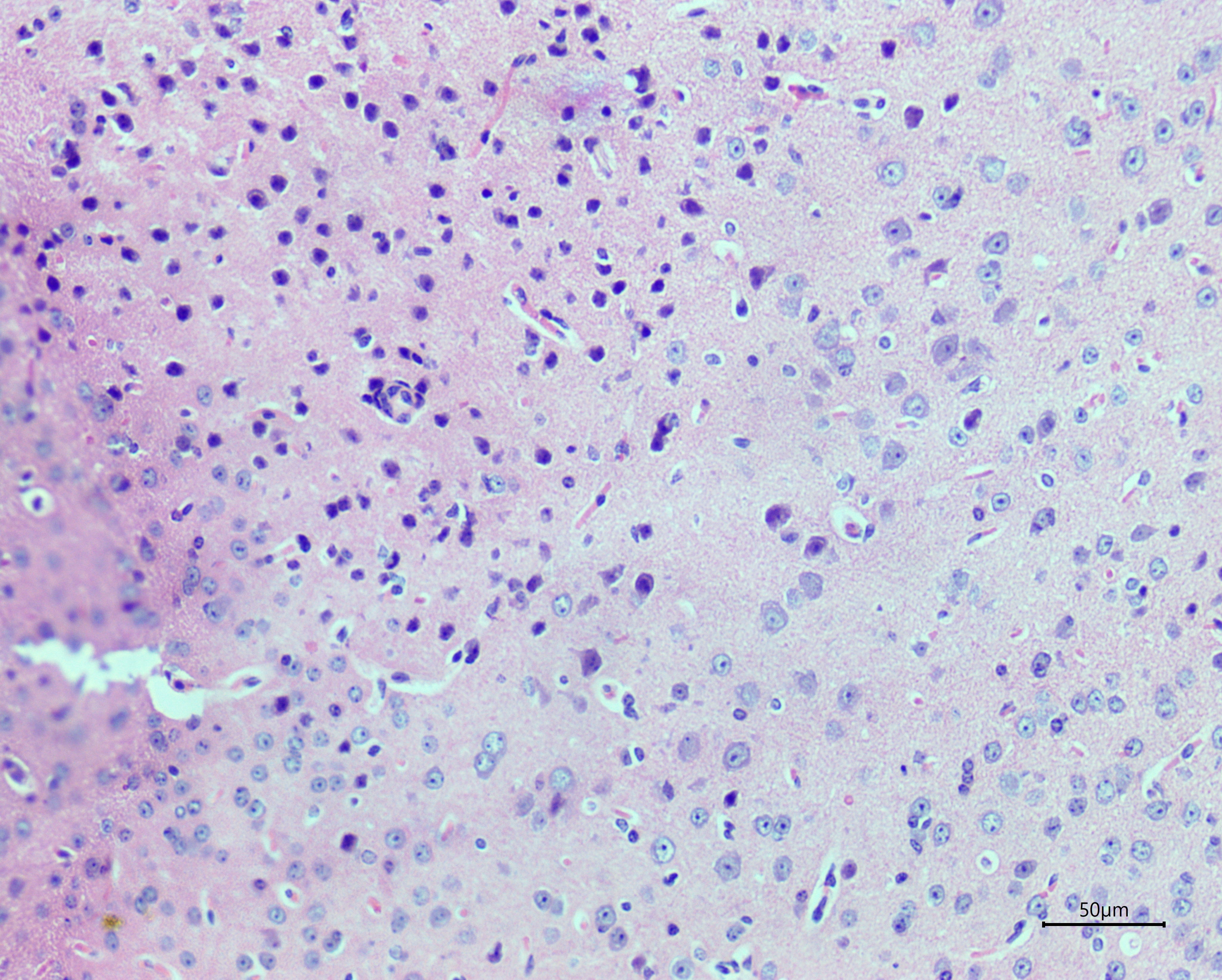

Supplement: S18 Fig — (JPG) [file pone.0250079.s018.jpg]

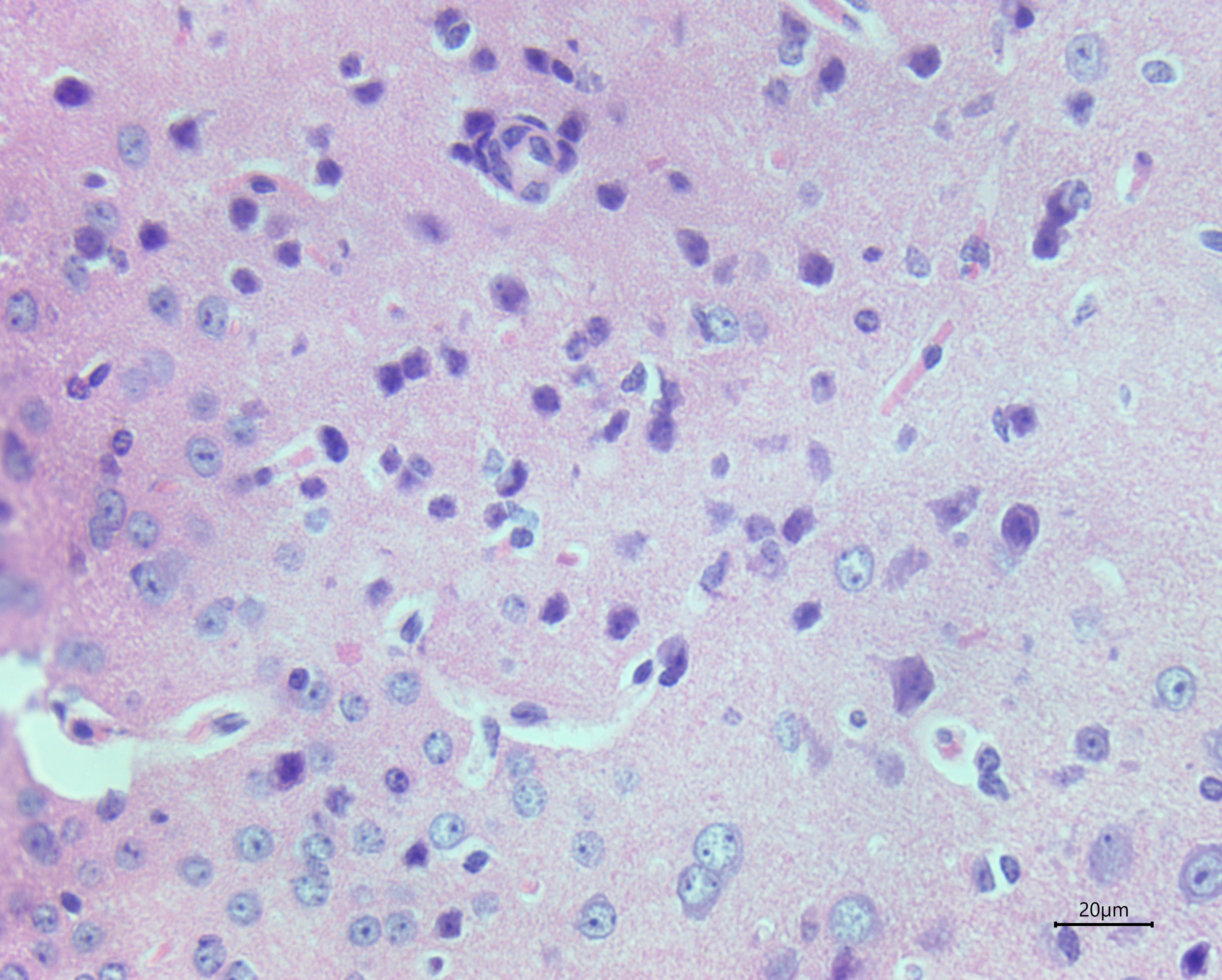

Supplement: S19 Fig — (JPG) [file pone.0250079.s019.jpg]

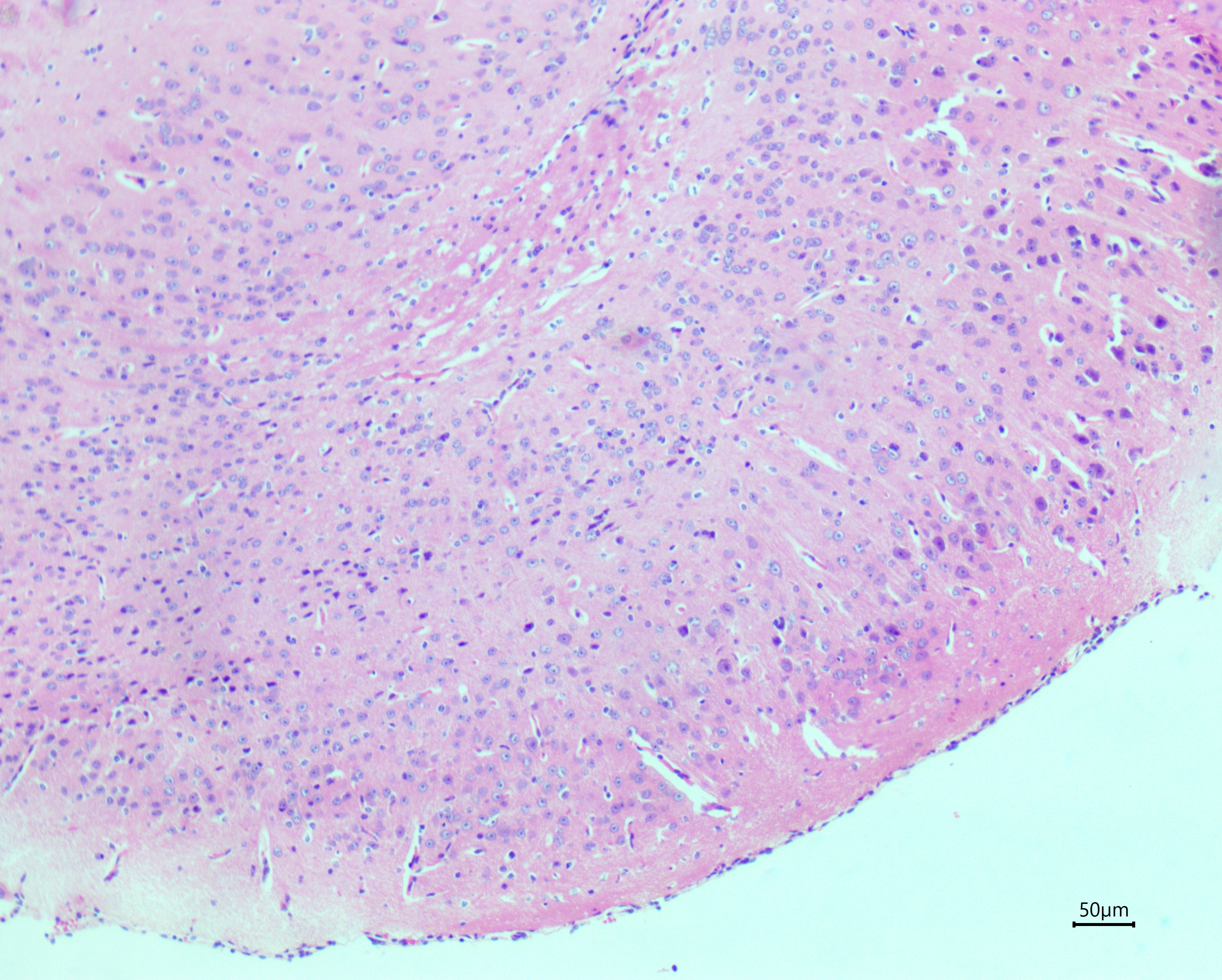

Supplement: S20 Fig — (JPG) [file pone.0250079.s020.jpg]

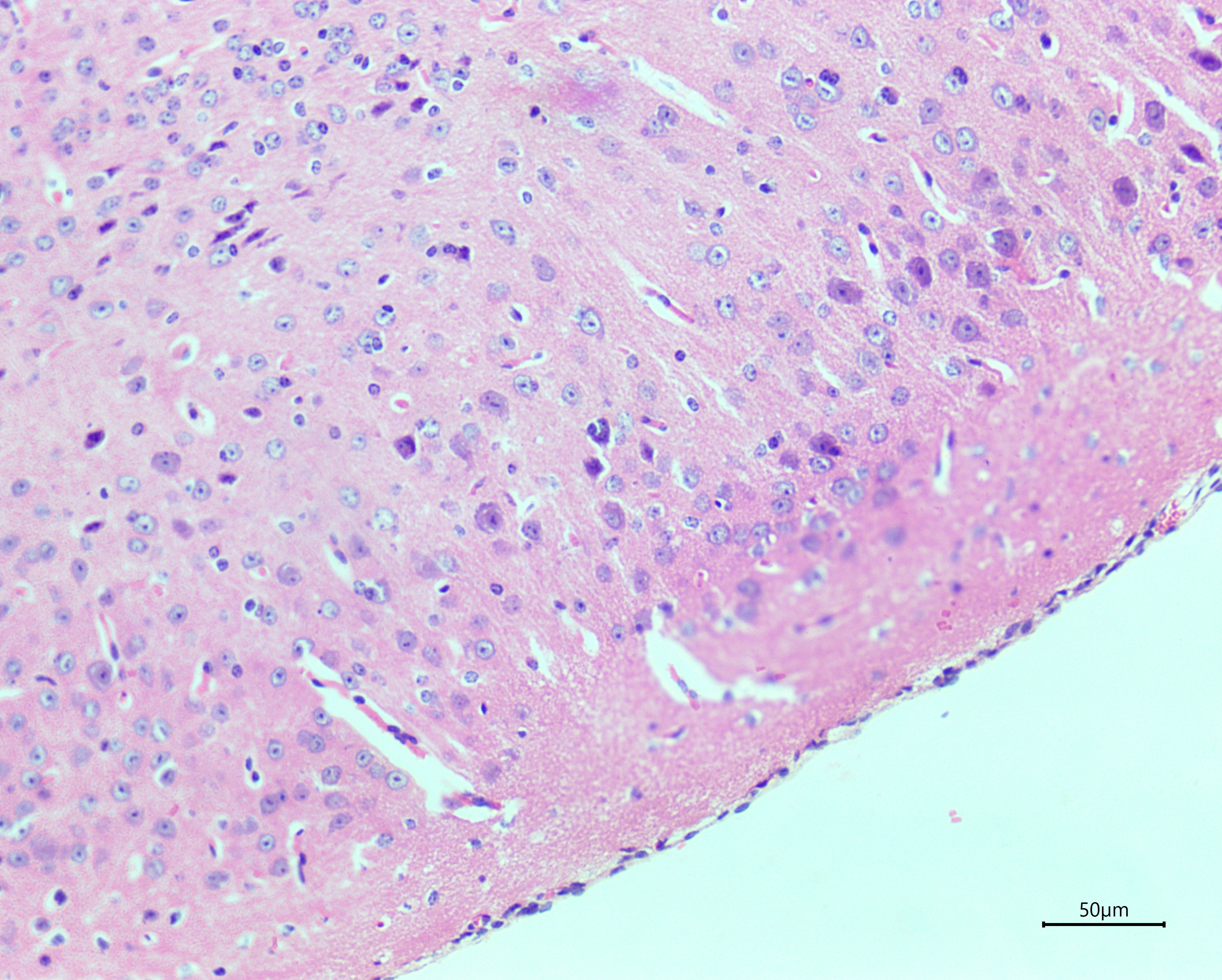

Supplement: S21 Fig — (JPG) [file pone.0250079.s021.jpg]
