# Supplementary material for: Rosuvastatin revert memory impairment and anxiogenic-like effect in mice infected with the chronic ME-49 strain of Toxoplasma gondii
Source: PLoS One. 2021 Apr 15;16(4):e0250079. doi: 10.1371/journal.pone.0250079 (PMC8049280; doi:10.1371/journal.pone.0250079)
Supplement: S1 Data — (DOCX) [file pone.0250079.s022.docx]

Data Table values used to build graphs and the means, standard deviations with other measures reported. The data represent the mean, standard deviation (SD), standard error of the mean (SEM) and number of animals per group (N).

|  | *BEHAVIOR* | *GROUP* | *TREATMENT* | *MEAN* | *SD* | *SEM* | *N* |
| --- | --- | --- | --- | --- | --- | --- | --- |
| *Memory Impairment* | **Short Memory** | **Non-Infected** | *Vehicle* | 0.653 | 0.040 | 0.013 | 10 |
|  |  |  | *Rosuvastatin* | 0.760 | 0.115 | 0.036 | 10 |
|  |  | **Infected** | *Vehicle* | 0.254 | 0.105 | 0.033 | 10 |
|  |  |  | *Rosuvastatin* | 0.675 | 0.056 | 0.018 | 10 |
|  | **Long Memory** | **Non-Infected** | *Vehicle* | 0.678 | 0.106 | 0.033 | 10 |
|  |  |  | *Rosuvastatin* | 0.722 | 0.098 | 0.031 | 10 |
|  |  | **Infected** | *Vehicle* | 0.244 | 0.166 | 0.052 | 10 |
|  |  |  | *Rosuvastatin* | 0.762 | 0.088 | 0.028 | 10 |
|  |  |  |  |  |  |  |  |
| *Open Field* | **Center Time (%)** | **Non-Infected** | *Vehicle* | 27.121 | 8.974 | 2.838 | 10 |
|  |  |  | *Rosuvastatin* | 27.274 | 8.817 | 2.788 | 10 |
|  |  | **Infected** | *Vehicle* | 14.880 | 7.852 | 2.483 | 10 |
|  |  |  | *Rosuvastatin* | 25.151 | 5.142 | 1.626 | 10 |
|  | **Central Locomotion** | **Non-Infected** | *Vehicle* | 10.000 | 3.651 | 1.155 | 10 |
|  |  |  | *Rosuvastatin* | 14.300 | 5.618 | 1.177 | 10 |
|  |  | **Infected** | *Vehicle* | 7.200 | 4.315 | 1.365 | 10 |
|  |  |  | *Rosuvastatin* | 13.100 | 2.183 | 0.690 | 10 |
|  | **Peripheral Locomotion** | **Non-Infected** | *Vehicle* | 39.200 | 10.218 | 3.231 | 10 |
|  |  |  | *Rosuvastatin* | 46.000 | 15.442 | 4.883 | 10 |
|  |  | **Infected** | *Vehicle* | 35.700 | 18.518 | 5.856 | 10 |
|  |  |  | *Rosuvastatin* | 44.600 | 10.013 | 3.166 | 10 |
|  | **Total Locomotion** | **Non-Infected** | *Vehicle* | 71.400 | 16.933 | 5.355 | 10 |
|  |  |  | *Rosuvastatin* | 94.600 | 20.935 | 6.620 | 10 |
|  |  | **Infected** | *Vehicle* | 70.300 | 33.136 | 10.479 | 10 |
|  |  |  | *Rosuvastatin* | 98.200 | 7.772 | 2.458 | 10 |
|  |  |  |  |  |  |  |  |

* In the article, the authors chose to build the graphs with the MEAN and the standard error of the mean (SEM).
