# Supplementary material for: Rosuvastatin revert memory impairment and anxiogenic-like effect in mice infected with the chronic ME-49 strain of Toxoplasma gondii
Source: PLoS One. 2021 Apr 15;16(4):e0250079. doi: 10.1371/journal.pone.0250079 (PMC8049280; doi:10.1371/journal.pone.0250079)
Supplement: S1 File — (DOCX) [file pone.0250079.s023.docx]

**List of images of histological sections of mice brains. Original magnification.**

- Imagens 1 – 2

Control group (non- infected / non-treated). Cerebral cortex with no abnormalities – 10x and 20x objectives respectively.

- Imagens 3 – 4

Infected control group (ME-49 strain / treated with vehicle). Inflammatory cell infiltration in the parenchyma, glial nodule, intense perivascular mononuclear infiltration – 10x and 20x objectives respectively.

- Imagens 5 – 6

Infected control group (ME-49 strain / treated with vehicle). Meninges with inflammation – 10x and 20x objectives respectively.

- Image 7

Infected control group (ME-49 strain / treated with vehicle). Gliosis area (lesion) – 10x objective.

- Image 8

Infected control group (ME-49 strain / treated with vehicle). Inflammatory cell infiltration, glial cell proliferation and perivascular cuff – 20x objective.

- Images 9 - 10

Infected control group (ME-49 strain / treated with vehicle). Diffuse inflammatory cell infiltration in tissue with focal hemorrhage – 10x and 20x objectives respectively.

- Images 11 – 13

Infected control group (ME-49 strain / treated with vehicle). *Toxoplasma gondii* cysts – 20x and 40x objectives.

- Images 14 – 15

Infected treated group (Rosuvastatin 40mg/Kg). Focal areas of gliosis indicating ancient injury, meninges with low inflammation and glial nodules. 10x and 20x objectives respectively.

- Images 16 – 17

Infected treated group (Rosuvastatin 40mg/Kg). Focal areas of gliosis indicating ancient injury. Blood vessel without perivascular infiltration. 10x and 20x objectives respectively.

- Images 18 – 19

Infected treated group (Rosuvastatin 40mg/Kg). Brain tissue with viable neurons. 20x and 40x objectives respectively.

- Images 20 – 21

Non-infected and treated control group (Rosuvastatin 40mg/kg). Normal brain tissue. 10x and 20x objectives respectively.
